# Kinetics of cooperative CO_2_ adsorption in diamine-appended variants of the metal–organic framework Mg_2_(dobpdc)†

**DOI:** 10.1039/d0sc01087a

**Published:** 2020-03-31

**Authors:** Jeffrey D. Martell, Phillip J. Milner, Rebecca L. Siegelman, Jeffrey R. Long

**Affiliations:** Department of Chemistry, University of California Berkeley CA 94720 USA jrlong@berkeley.edu; Department of Chemical and Biomolecular Engineering, University of California Berkeley CA 94720 USA; Materials Sciences Division, Lawrence Berkeley National Laboratory Berkeley CA 94720 USA

## Abstract

Carbon capture and sequestration is a key element of global initiatives to minimize anthropogenic greenhouse gas emissions. Although many investigations of new candidate CO_2_ capture materials focus on equilibrium adsorption properties, it is also critical to consider adsorption/desorption kinetics when evaluating adsorbent performance. Diamine-appended variants of the metal–organic framework Mg_2_(dobpdc) (dobpdc^4−^ = 4,4′-dioxidobiphenyl-3,3′-dicarboxylate) are promising materials for CO_2_ capture because of their cooperative chemisorption mechanism and associated step-shaped equilibrium isotherms, which enable large working capacities to be accessed with small temperature swings. However, the adsorption/desorption kinetics of these unique materials remain understudied. More generally, despite the necessity of kinetics characterization to advance adsorbents toward commercial separations, detailed kinetic studies of metal–organic framework-based gas separations remain rare. Here, we systematically investigate the CO_2_ adsorption kinetics of diamine-appended Mg_2_(dobpdc) variants using a thermogravimetric analysis (TGA) assay. In particular, we examine the effects of diamine structure, temperature, and partial pressure on CO_2_ adsorption and desorption kinetics. Importantly, most diamine-appended Mg_2_(dobpdc) variants exhibit an induction period prior to reaching the maximum rate of CO_2_ adsorption, which we attribute to their unique cooperative chemisorption mechanism. In addition, these materials exhibit inverse Arrhenius behavior, displaying faster adsorption kinetics and shorter induction periods at lower temperatures. Using the Avrami model for nucleation and growth kinetics, we determine rate constants for CO_2_ adsorption and quantitatively compare rate constants among different diamine-appended variants. Overall, these results provide guidelines for optimizing adsorbent design to facilitate CO_2_ capture from diverse target streams and highlight kinetic phenomena relevant for other materials in which cooperative chemisorption mechanisms are operative.

## Abstract 2

An in-depth investigation of the CO_2_ adsorption kinetics of a promising class of cooperative carbon capture materials offers new insight into their structure-performance properties.

## Graphical abstract

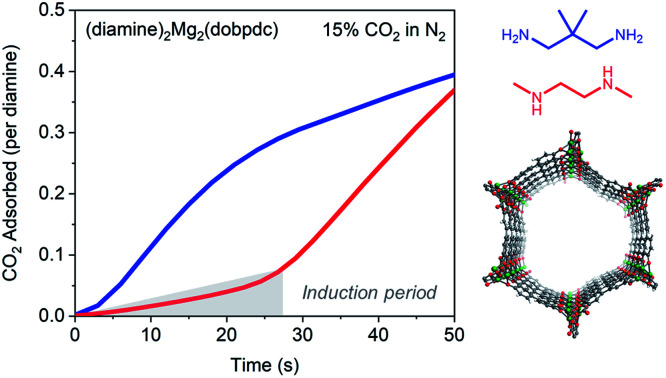

## Introduction

Steadily rising atmospheric CO_2_ levels and the associated increase in average global temperatures have created an urgent need to curb anthropogenic CO_2_ emissions.^1^ While long-term solutions to this challenge necessitate a shift to renewable energy sources, fossil fuels will continue to supply a major portion of global energy in the near future.^2^ One proposed strategy to mitigate atmospheric CO_2_ emissions in the short term is carbon capture and sequestration from major point sources, such as power plants.^3^ To realize this strategy, carbon capture materials are needed that possess high selectivities and CO_2_ capacities, as well as minimal energy requirements for CO_2_ desorption. Rapid kinetics to bind and release CO_2_ are also critical, because they can dictate bed utilization and thus impact the cost and efficiency of a carbon capture process.^4^

Aqueous solutions of organic amines are a mature CO_2_ capture technology, but they suffer from numerous drawbacks. For example, solutions with relatively low amine concentrations are necessary to minimize corrosive effects, thereby decreasing the solution CO_2_ absorption capacities and increasing the energy required to heat the absorbent during temperature-swing cycling.^5^ As a result, the implementation of aqueous amine scrubbers in a power plant places a parasitic load of 25–30% on the net power output.^6^ Furthermore, aqueous amine solutions are prone to thermal and oxidative degradation.^3^ As an alternative, porous solid adsorbents have been proposed as capture materials, owing to their lower heat capacities and high surface areas, which create the potential for high adsorption capacities and more efficient adsorption–desorption cycling. Nevertheless, many of these solid adsorbents fail to capture CO_2_ selectively from humid gas streams.^7^

**Fig. 1:**
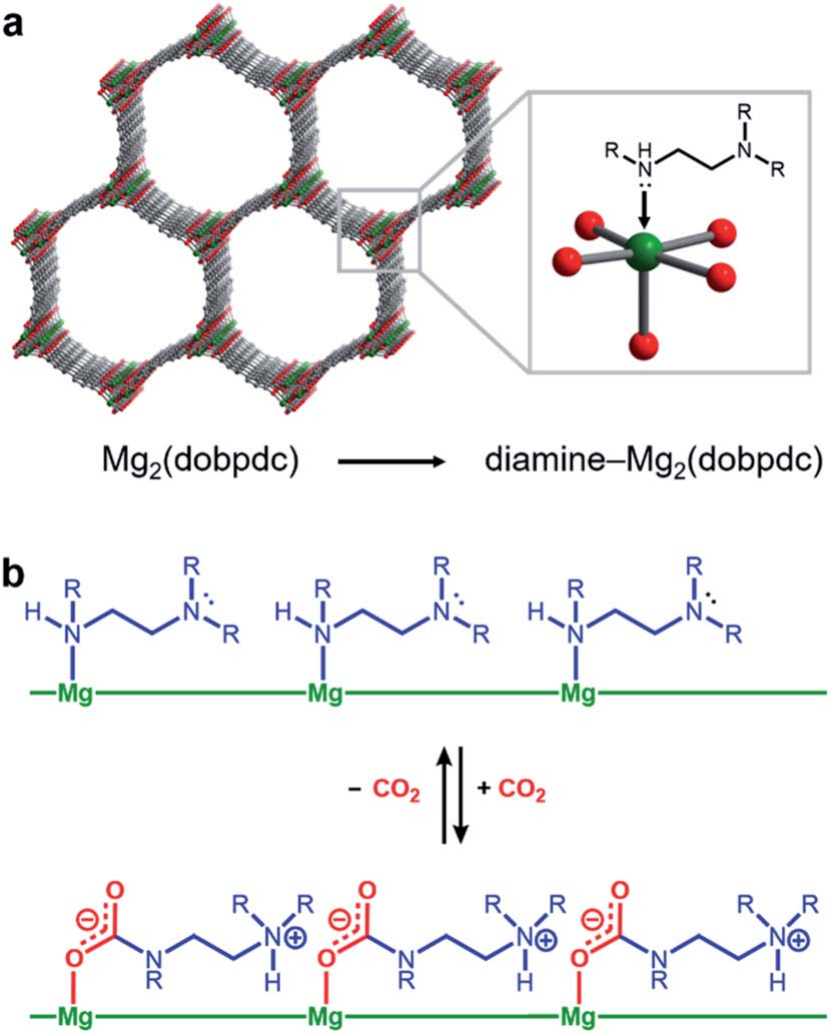
Structure of diamine-appended variants of the metal–organic framework Mg_2_(dobpdc). (a) Diamines are appended post-synthetically to the open Mg^2+^ sites of the framework. (b) Schematic representation of the resulting adsorbents capturing CO_2_ cooperatively through the formation of ammonium carbamate chains.

Amine-functionalized solid adsorbents combine the advantages of aqueous amine solutions and porous solid adsorbents. Examples of these materials include amine-functionalized silicas,^8–11^ porous polymers,^12,13^ zeolites,^14,15^ and metal–organic frameworks.^16–20^ Owing to their high crystallinity and chemical adjustability, metal–organic frameworks possess ordered structures that can be tailored with respect to pore size, shape, and chemical environment. In particular, amine functionalities can be incorporated within the organic linkers of these materials, both during framework synthesis^21^ or through post-synthetic modification.^16–20,22^ Additionally, the high internal surface areas accessible with metal–organic frameworks can allow for rapid diffusion of CO_2_ through the pores.^23^ Diamine-appended variants of the metal–organic framework Mg_2_(dobpdc)^24–31^ (dobpdc^4−^ = 4,4′-dioxidobiphenyl-3,3′-dicarboxylate) represent a particularly promising class of amine-functionalized frameworks, as they readily adsorb CO_2_ through a chemically-specific, cooperative mechanism (Fig. 1).^25^ Here, the metal-bound amine reacts covalently with CO_2_ to generate a carbamate while the pendent amine is concomitantly protonated. This process propagates through the material to yield chains of ammonium carbamate stabilized through ionic interactions along the pore axis. As a result of this unique capture mechanism, diamine-appended variants of Mg_2_(dobpdc) exhibit step-shaped CO_2_ adsorption profiles, which give rise to large CO_2_ cycling capacities that are accessible with relatively small temperature swings.^25^ Importantly, by varying the metal cation,^25^ diamine,^26–28^ or organic linker,^28^ the adsorption step position can be tuned in pressure by over five orders of magnitude (from ∼10^−5^ to ∼1 bar at 40 °C) to enable the precise targeting of specific CO_2_ separation conditions. Moreover, these materials have also been shown to maintain high CO_2_ working capacities after 1000 adsorption/desorption cycles under humid gas streams.^27^

While the thermodynamics of CO_2_ capture in diamine-appended variants of Mg_2_(dobpdc) are promising for numerous carbon capture applications, the kinetics also play a crucial role in the practical application of these materials. For porous solid adsorbents, small-scale breakthrough experiments are often used to simulate a fixed-bed adsorption process. In these experiments, shaped particles of the adsorbent are packed into a column, a CO_2_-containing gas stream is fed through the inlet, and the outlet composition and flow rate are measured as a function of time until CO_2_ “breaks through.” Multiple kinetic parameters can influence the performance of an adsorbent in a fixed bed, including interparticle, intraparticle, and micropore diffusional resistances, as well as potential reaction limitations for amine-based chemisorption of CO_2_. Critically, the overall CO_2_ adsorption kinetics must be sufficiently fast to maximize bed utilization in the process. Promising initial results have been obtained for diamine-appended Mg_2_(dobpdc) variants in gram-scale breakthrough experiments for CO_2_ capture from simulated coal flue gas (15% CO_2_ in N_2_) under both dry and humid conditions.^27^ In addition, rapid cycle times have been employed for diamine-appended Mg_2_(dobpdc) variants in simulated temperature-swing experiments under 15% CO_2_ with a pure CO_2_ purge, and here cycle times were limited only by the temperature ramp rate of the thermogravimetric analyzer.^27,28^

Given the promise of diamine-appended Mg_2_(dobpdc) for CO_2_ capture applications, a detailed analysis of the kinetics of CO_2_ adsorption in these materials is necessary for optimal process implementation. Toward this end, we herein utilize thermogravimetric analysis (TGA) to systematically investigate the dry CO_2_ adsorption kinetics in diamine-appended variants of Mg_2_(dobpdc) under a range of adsorption conditions. Our results demonstrate the influence of adsorption temperature, CO_2_ concentration, and diamine structure on the rate of adsorption. On the basis of these correlations, we conclude with guidelines for the optimization of adsorbent structure and process parameters in CO_2_ capture applications.

## Results and discussion

### Experimental setup for a thermogravimetric assay to study CO_2_ adsorption kinetics

Diamine-appended variants of Mg_2_(dobpdc) were synthesized using our previously reported procedure.^26^ Specifically, methanol-solvated Mg_2_(dobpdc) was soaked in a 20% (v/v) toluene solution of the diamine of interest, and subsequent filtration, washing with toluene, and activation yielded adsorbents with one diamine per metal site. To ensure internal consistency for this study, all experiments were performed with the same batch of Mg_2_(dobpdc). The rod-shaped crystallites in this batch exhibited an aspect ratio of ∼10 and were heterogeneous in size, with widths ranging from ∼60 nm to >1 μm (Fig. S1†). Measurements in this study were carried out on as-synthesized diamine–Mg_2_(dobpdc) samples in powder form; future studies will explore adsorption/desorption kinetics in structured forms of these materials.

**Fig. 2:**
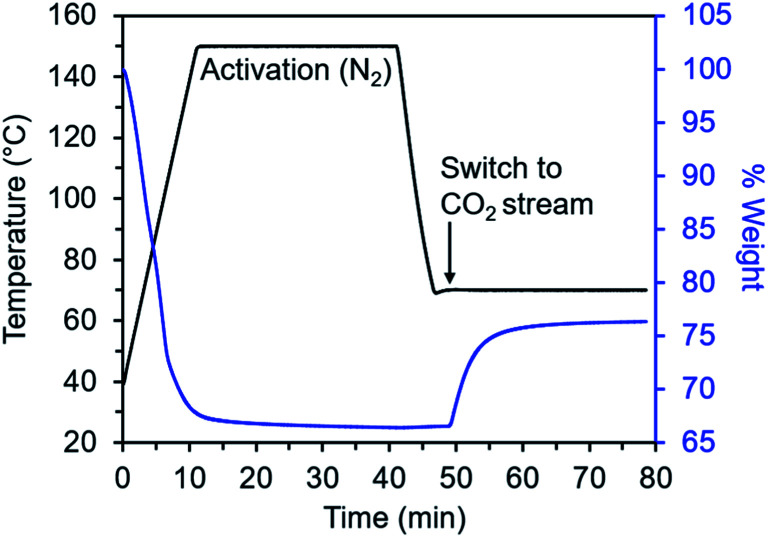
Overview of TGA adsorption kinetics assay. The diamine-appended metal–organic framework sample is activated within a thermogravimetric analyzer under flowing N_2_ to remove solvent molecules and non-metal-bound diamines from the pores. The sample is then cooled under N_2_ to the temperature of interest, the gas flow is switched to a CO_2_-containing stream, and CO_2_ adsorption is monitored as a function of time.

We developed a TGA assay at atmospheric pressure to evaluate the CO_2_ adsorption kinetics of diamine-appended Mg_2_(dobpdc). In this assay, samples were first activated in the TGA furnace under flowing N_2_ to remove any captured CO_2_, solvent, or excess diamine present in the pores. Next, samples were cooled to a temperature of interest, the gas flow was switched to a CO_2_-containing stream, and the change in mass was monitored as a function of time (Fig. 2). Because N_2_ adsorption is negligible at and above room temperature in these materials,^26,27^ we approximated that all mass increase was due to CO_2_ adsorption. In addition, mass changes due to buoyancy effects upon switching the gas stream from N_2_ to CO_2_ were negligible compared to the mass of CO_2_ adsorbed by the standard sample size of 3 mg of diamine–Mg_2_(dobpdc) used in this work (Fig. S2†).

This TGA-based assay, which has been reported for fundamental kinetics characterization of other solid CO_2_ adsorbents,^11,32–35^ is advantageous given its simple setup, the small sample mass requirement, and the fact that adsorbents can be activated *in situ* and rapidly tested under many temperatures and partial pressures of CO_2_. However, this assay also has some limitations. For example, because TGA detects gas adsorption through a change in sample mass, it cannot discriminate between CO_2_, H_2_O, and N_2_ adsorption (although, as mentioned above, N_2_ adsorption is negligible for diamine-appended Mg_2_(dobpdc) variants under the conditions relevant for CO_2_ chemisorption). As a result, determining CO_2_ adsorption kinetics under humid conditions remains challenging, and here we discuss only dry adsorption/desorption kinetics profiles. In addition, the first few points of kinetics data from a TGA experiment are obscured by exchange of the initial gas in the furnace with the CO_2_-containing stream in adsorption experiments or the N_2_ purge stream in desorption experiments.

As has been previously noted,^11^ differences in flow rate and sample mass can also greatly affect TGA adsorption kinetics profiles. For example, in the case of m-2-m–Mg_2_(dobpdc) (m-2-m = *N*,*N*′-dimethylethylenediamine), we found that the sample mass has a substantial impact on adsorption kinetics from a simulated coal flue gas stream of 15% CO_2_ in N_2_,^36^ with larger samples displaying slower overall adsorption kinetics (Fig. S3†). Therefore, to maintain consistency across all samples in this study, we used a sample mass of 3 mg and ensured the powder was evenly distributed across the surface of the TGA pan. Also consistent with previous reports, faster adsorption kinetics were observed with faster flow rates (Fig. S3†). This flow rate effect could be due to the time required to completely exchange the initial gas in the furnace and/or to the observed kinetics being influenced by a mass transfer resistance related to diffusion. As a result, the kinetics of adsorption presented in this work likely represent lower bounds on the intrinsic adsorption kinetics of these materials. For the fastest flow rates tested (>100 mL min^−1^), adsorption was essentially complete within the first few data points collected on the TGA, making it difficult to quantitatively compare the kinetics among different adsorbents under these conditions. We therefore used a consistent flow rate of 25 mL min^−1^ for all experiments to facilitate quantitative comparisons among the diamine-appended variants. Under these conditions, a consistent delay of 19 s was observed before the sample mass increased, after switching the TGA valving to the analysis gas. This delay time corresponds to the time required for CO_2_ to reach the sample in the TGA furnace. Additionally, given a flow rate of 25 mL min^−1^, we approximate that at least 14–23 s are required at temperatures ranging from 120 to 30 °C for complete exchange of gases in the TGA furnace, beyond the initial 19 s delay (Table S1†). By accounting for these considerations in the measurements detailed below, we were able to compare the effects of temperature, CO_2_ partial pressure, and diamine structure on the CO_2_ adsorption kinetics of these materials.

### Adsorption kinetics and temperature dependence for m-2-m–Mg_2_(dobpdc)

**Fig. 3:**
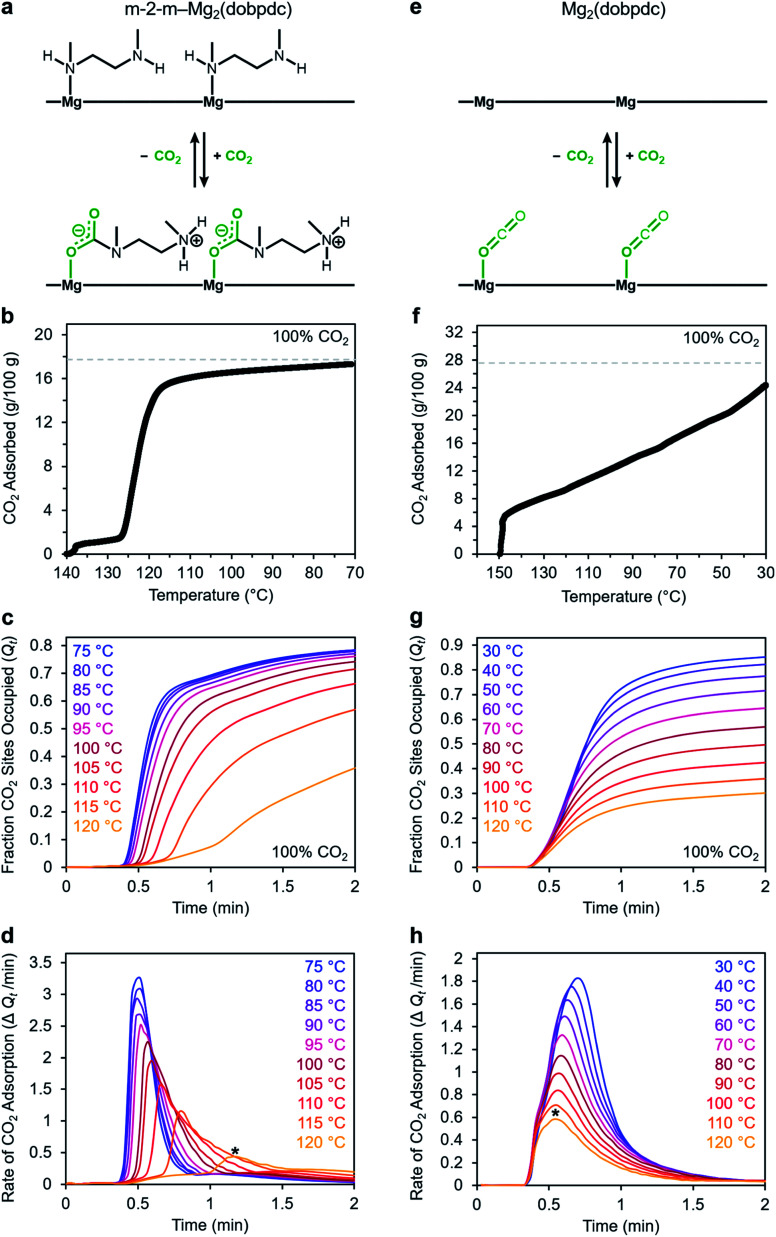
Kinetics profiles for adsorption of CO_2_ m-2-m–Mg_2_(dobpdc) (left column) and Mg_2_(dobpdc) (right column). Panels (a) and (e): schematic representations of CO_2_ adsorption mechanisms for m-2-m–Mg_2_(dobpdc) and Mg_2_(dobpdc), respectively. Panels (b) and (f): pure CO_2_ adsorption isobars for m-2-m–Mg_2_(dobpdc) and Mg_2_(dobpdc), respectively. Samples were activated under flowing N_2_ at 140 °C for 30 min (b) or at 300 °C for 15 min (f). Cooling ramp rate: 1 °C min^−1^. The gray dotted lines represent the adsorption capacity corresponding to either 1 CO_2_ per diamine in m-2-m–Mg_2_(dobpdc) (17.8 g/100 g) (b), or 1 CO_2_ per open metal coordination site in Mg_2_(dobpdc) (27.6 g/100 g) (f). Panels (c) and (g): pure CO_2_ adsorption *vs.* time plots for m-2-m–Mg_2_(dobpdc) and Mg_2_(dobpdc), respectively. Samples were activated under flowing N_2_ at 130 °C for 5 min (c) or at 310 °C for 5 min (g) prior to CO_2_ adsorption at the indicated temperatures. “Fraction CO_2_ sites occupied”, or *Q*_*t*_, indicates the ratio of adsorbed CO_2_ molecules to m-2-m in the material (c) or the ratio of adsorbed CO_2_ molecules to Mg^2+^ sites in the material (g). Panels (d) and (h): rate of CO_2_ adsorption *vs.* time corresponding to the data shown in (c) and (g), respectively. The asterisks in panels (d) and (h) indicate the times at which the maximum rate of adsorption was observed during the 120 °C adsorption experiments. In panels c–d and g–h, a 19 s delay is observed prior to the sample mass increasing, corresponding to the time required for CO_2_ to reach the sample in the furnace.

We utilized our optimized TGA assay to characterize the CO_2_ adsorption kinetics of m-2-m–Mg_2_(dobpdc), the first reported diamine-appended variant of Mg_2_(dobpdc),^24^ at selected temperatures. Extensive gas adsorption, structural, and spectroscopic data have been previously reported for this material,^24–26^ and an overview of the adsorption mechanism is shown in Fig. 3a. We first investigated the CO_2_ adsorption kinetics of m-2-m–Mg_2_(dobpdc) from a pure CO_2_ stream at atmospheric pressure over a range of temperatures below the adsorption step temperature (*T*_step_) of 127 °C,^26^ defined here as the onset of the step-shaped adsorption isobar (Fig. 3b). Rapid uptake of CO_2_ was observed below *T*_step_, as is evident in a plot of fraction of diamine sites occupied (*Q*_*t*_) *vs.* time (Fig. 3c). Interestingly, adsorption of CO_2_ is faster at lower temperatures and follows an inverse Arrhenius behavior (Fig. 3d). Such inverse Arrhenius behavior has been observed previously for a polyethylenimine-appended mesoporous silica^11^ and amine-functionalized carbon nanotubes,^37^ whereas normal Arrhenius behavior has been observed for other amine-appended solid adsorbents.^34,35,38^ Our results suggest that although the *equilibrium* capacity of m-2-m–Mg_2_(dobpdc) is relatively insensitive to temperature below *T*_step_,^26^ lower adsorption temperatures promote more rapid saturation with CO_2_. A more in-depth discussion of this phenomenon is provided below (see “Inverse Arrhenius behavior”).

In addition to this inverse Arrhenius behavior, we also observed an induction period between the time at which the mass begins to increase, which corresponds to CO_2_ first entering the furnace, and the time associated with the fastest rate of CO_2_ uptake (Fig. 3d). This effect is particularly pronounced at temperatures close to *T*_step_. To elucidate whether the observed induction period is intrinsic to the CO_2_ adsorption kinetics of m-2-m–Mg_2_(dobpdc) or is an artifact of the experimental setup, we investigated CO_2_ adsorption in the bare framework material with no appended diamines, Mg_2_(dobpdc) (Fig. 3e and f). In Mg_2_(dobpdc), CO_2_ binds to open metal coordination sites exposed upon solvent removal from the framework, leading to a typical Langmuir adsorption profile (Fig. 3e).^24^ Importantly, because CO_2_ adsorption in Mg_2_(dobpdc) does not involve a chemical reaction, the adsorption kinetics are likely diffusion-limited. Consistently, Mg_2_(dobpdc) reaches its equilibrium CO_2_ adsorption capacity within a similarly short time for all temperatures examined (Fig. 3g and h), with the maximum rate of adsorption occurring at earlier times as the temperature is increased, in stark contrast to the behavior exhibited by m-2-m–Mg_2_(dobpdc). For example, at 120 °C, approximately 51 s elapses before the maximum rate of adsorption is achieved in m-2-m–Mg_2_(dobpdc), whereas the maximum rate is reached in only 14 s for bare Mg_2_(dobpdc) under the same conditions (see asterisks in Fig. 3d and h). Note that comparison of the absolute rate of adsorption is complicated by the lower equilibrium capacity of Mg_2_(dobpdc) at the low partial pressures present in the TGA furnace during the initial mixing period (Fig. S4†). Furthermore, the apparent kinetics of Mg_2_(dobpdc) include competition of N_2_ and CO_2_ for the same binding sites, and thus CO_2_ adsorption requires displacement of any adsorbed N_2_ molecules. Nevertheless, only a small fraction of metal sites (∼2–10%) is expected to be occupied by N_2_ under the conditions examined here, based on previously reported N_2_ adsorption isotherms for the isoreticular smaller-pore framework material Mg_2_(dobdc).^39^

**Fig. 4:**
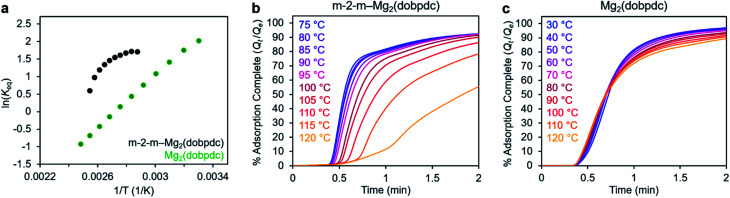
(a) Van't Hoff plot depicting the equilibrium CO_2_ adsorption capacity with varying temperature for m-2-m–Mg_2_(dobpdc) and Mg_2_(dobpdc) from a 100% CO_2_ stream (based on the data shown in Fig. 3c and g). *K*_eq_ is defined as *Q*_e_/[(*Q*_max_ − *Q*_e_)*p*CO_2_], where *Q*_e_ is the equilibrium adsorption capacity, *Q*_max_ is the theoretical maximum CO_2_ adsorption capacity, and *p*CO_2_ is the partial pressure of CO_2_, defined as *p*/*p*_0_, with *p* = *p*_0_ = 1 atm. We note that this van't Hoff analysis assumes an equilibrium exchange. Although the chemisorbed CO_2_ in saturated ammonium carbamate chains does not appreciably exchange with free CO_2_,^52^ the changes in adsorption capacity for m-2-m–Mg_2_(dobpdc) at temperatures below the step are likely due primarily to CO_2_ physisorption, which should be reversible. The enthalpy of CO_2_ adsorption, Δ*h*, can be estimated for bare Mg_2_(dobpdc) based on the slope of a linear fit to the data points (Fig. S7†). (b) and (c): plots of % adsorption complete (*Q*_*t*_/*Q*_e_) *vs.* time corresponding to the data shown in Fig. 3c and g, respectively. Adsorption was monitored for 30 min (b) or 10 min (c) to confirm mass equilibration, but only the first 2 min of adsorption are shown.

In addition to the marked differences in the kinetics of CO_2_ adsorption, the bare framework Mg_2_(dobpdc) and amine-appended m-2-m–Mg_2_(dobpdc) also exhibit distinct trends in their variable–temperature equilibrium CO_2_ adsorption capacities (*Q*_e_). Whereas the CO_2_ saturation capacity of m-2-m–Mg_2_(dobpdc) is similar across nearly all temperatures investigated, decreasing only near *T*_step_ (Fig. 3b), the capacity of Mg_2_(dobpdc) varies substantially with temperature (Fig. 3f). This difference arises due to the different adsorption profiles of these two materials and is best demonstrated through a van't Hoff plot (Fig. 4a). To quantify the kinetics in these two materials while accounting for variations in equilibrium capacity, we also analyzed the percent of adsorption complete (*Q*_*t*_/*Q*_e_) *vs.* time (Fig. 4b and c). This analysis again reveals that adsorption is faster at lower temperatures for m-2-m–Mg_2_(dobpdc), with induction periods observed at temperatures close to *T*_step_. In contrast, the adsorption profiles are similar at all temperatures for Mg_2_(dobpdc), with the highest temperatures exhibiting the fastest initial progress toward equilibrium. Overall, these findings corroborate that the chemisorptive mechanism operational in m-2-m–Mg_2_(dobpdc) leads to an unusual induction period that is not observed in the physisorptive mechanism in Mg_2_(dobpdc).

**Fig. 5:**
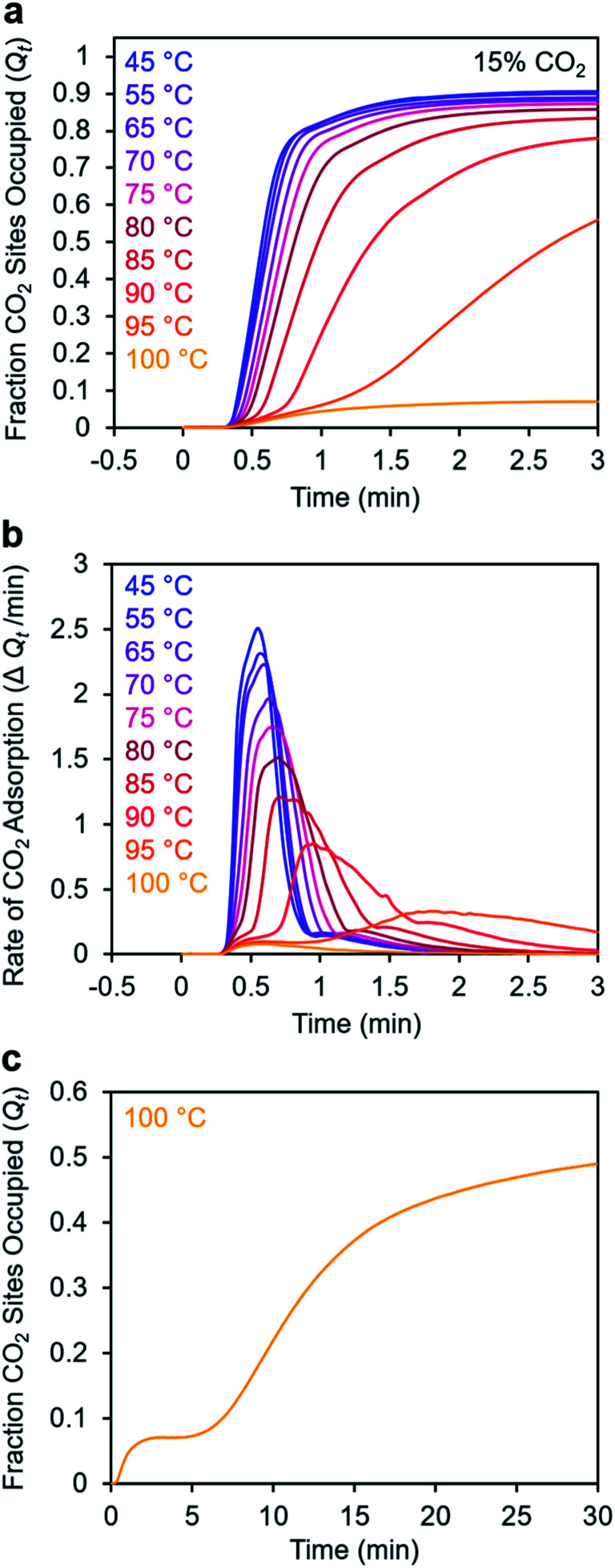
Adsorption kinetics from a 15% CO_2_ in N_2_ stream for m-2-m–Mg_2_(dobpdc). (a) Fraction of CO_2_ sites occupied *vs.* time. The sample was activated at 130 °C for 5 min under flowing N_2_ prior to CO_2_ adsorption at the indicated temperatures. (b) Rate of CO_2_ adsorption *vs.* time corresponding to the data shown in (a). (c) Data collected at 100 °C (as shown in (a)) with the *x*-axis extended. A delay of approximately 10 min was observed before the maximum rate of adsorption was reached. In this plot, data are normalized such that *t* = 0 is after the 19 s delay prior to CO_2_ reaching the sample in the furnace.

To further understand the induction period exhibited by m-2-m–Mg_2_(dobpdc), we characterized the adsorption kinetics from a 15% CO_2_ stream (Fig. 5), corresponding to the approximate partial pressure of CO_2_ in coal flue gas.^36^ In this case, the investigated temperature range (45–100 °C) is lower than that used in the experiments with 100% CO_2_ (75–120 °C), reflecting the lower adsorption step temperature under 15% CO_2_ (102 °C) compared to 100% CO_2_ (127 °C) (Fig. S8†). At temperatures below 75 °C, adsorption was nearly complete in less than 1 min. At temperatures near *T*_step_, however, the induction period was even more pronounced than in the analogous experiments with 100% CO_2_ (Fig. 5b*vs.*Fig. 3d). The effect was particularly dramatic at 100 °C (Fig. 5c)—a small amount of CO_2_ was rapidly adsorbed (6% of the diamine sites occupied within 1 min), followed by a period of 4 min during which almost no additional adsorption occurred. After 5 min, the rate of CO_2_ adsorption accelerated, and a substantial CO_2_ occupancy was ultimately reached, corresponding to occupation of approximately half of the diamine sites. The fast capture of CO_2_ at ∼6% of the m-2-m sites, as shown in Fig. 5c, is consistent with the previous finding that m-2-m–Mg_2_(dobpdc) adsorbs a small amount of CO_2_ even at pressures below the step.^24,25^ Spectroscopic studies indicate that this pre-step chemisorption arises due to ammonium carbamate species that form without metal–amine insertion.^7^ We hypothesize that the pre-step ammonium carbamate species forms rapidly, followed by slower cooperative adsorption of additional CO_2_*via* the formation of metal-bound ammonium carbamate chains. Qualitatively similar results were obtained using a 5% CO_2_ stream, for which the induction period is even more pronounced (Fig. S6†).

Taken together, these results suggest that the sigmoidal kinetics profile of m-2-m–Mg_2_(dobpdc) is directly related to its cooperative CO_2_ adsorption mechanism. This material exhibits a high degree of cooperativity in equilibrium gas adsorption isotherms, with its Hill coefficient of ∼11 indicating that, from a thermodynamic standpoint, capturing one CO_2_ molecule facilitates the adsorption of subsequent CO_2_ molecules.^25^ The pronounced sigmoidal kinetics profile exhibited by m-2-m–Mg_2_(dobpdc) likewise suggests that initial capture of a CO_2_ molecule also enhances the adsorption kinetics of subsequent CO_2_ molecules. Similar sigmoidal kinetic profiles have been reported previously for autocatalytic^40^ and autoinductive^41^ chemical reactions. While a recent TGA-based assay previously revealed that a polyethylenimine-appended mesoporous silica also exhibited sigmoidal CO_2_ adsorption kinetics,^11^ amine-impregnated clays did not exhibit sigmoidal uptake kinetics when evaluated using comparable TGA equipment and procedures.^34^ These precedents establish that not all amine-based CO_2_ adsorbents exhibit sigmoidal kinetics. As noted in a recent study,^42^ a long induction period is undesirable for implementation in a practical process because it can lead to lower bed utilization. Specifically, in breakthrough experiments simulating direct air capture with m-2-m–Mg_2_(dobpdc), it was found that after rapid partial breakthrough of CO_2_, the CO_2_ concentration at the outlet *decreased* before full breakthrough eventually occurred, consistent with a delayed onset of adsorption.^42^ Hence, it is critical to identify appropriate diamine variants and/or CO_2_ adsorption conditions that minimize this induction period (see below).

### Avrami model of the adsorption kinetics of m-2-m–Mg_2_(dobpdc)

We next sought to model the CO_2_ adsorption kinetics of m-2-m–Mg_2_(dobpdc) from a gas stream of 15% CO_2_ in N_2_. The fraction of sites occupied at time *t* (*Q*_*t*_) was fit at each temperature using either a pseudo-first order model (eqn (1)) or Avrami's kinetics model (eqn (2)), which was originally developed to model nucleation-growth kinetics^43^ and has recently found application as a model for chemisorption in an amine-appended mesoporous silica.^11^1
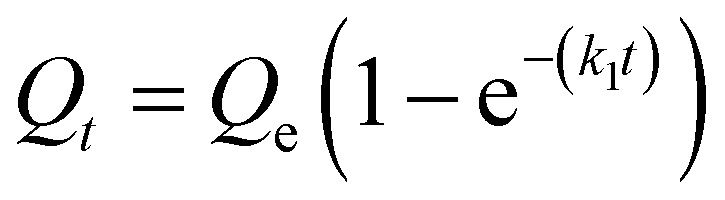
2
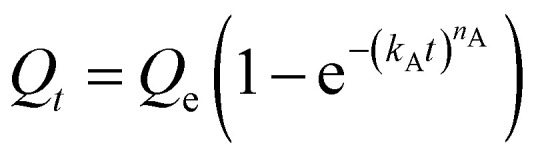


**Fig. 6:**
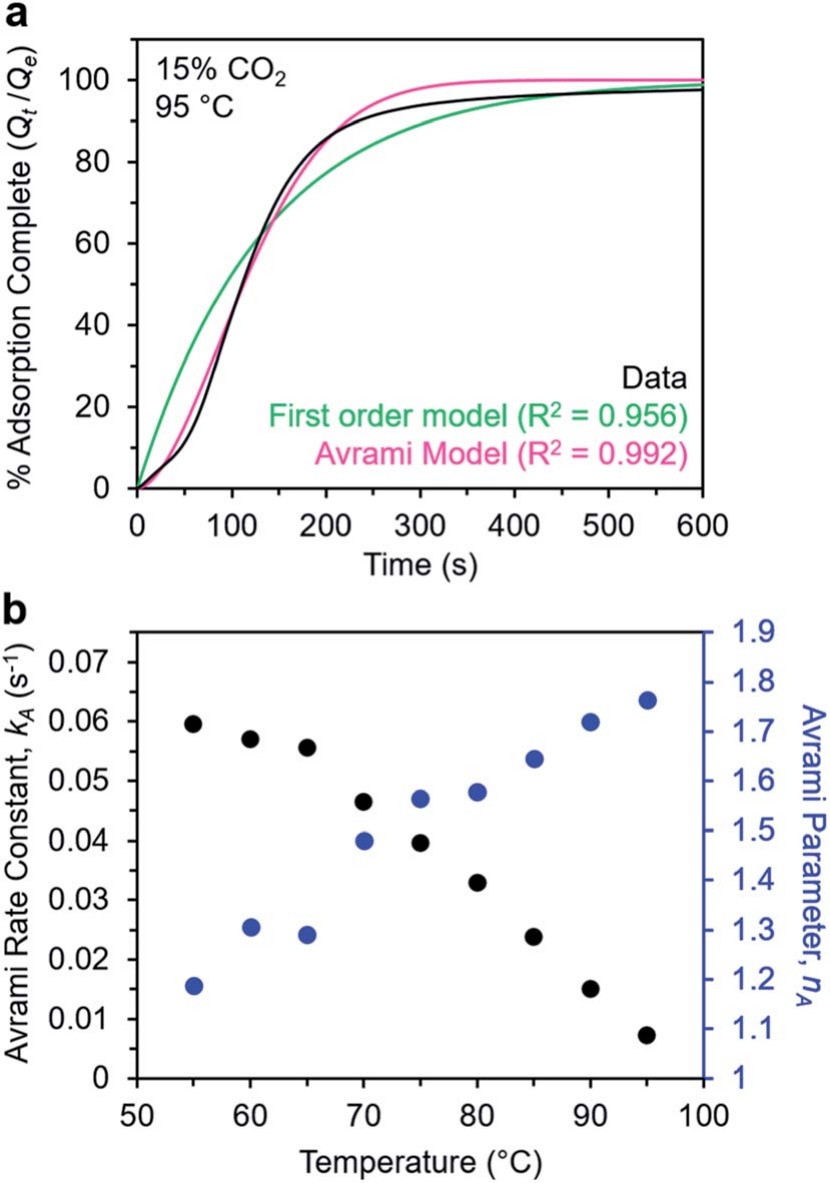
Models for the kinetics of CO_2_ adsorption from a 15% stream by m-2-m–Mg_2_(dobpdc). (a) CO_2_ adsorption *vs.* time (black) at 95 °C from a 15% CO_2_ stream for m-2-m–Mg_2_(dobpdc). Pseudo-first order and Avrami fits to the data are shown as green and magenta lines, respectively. In this plot, data are normalized such that *t* = 0 occurs after the 19 s delay prior to CO_2_ reaching the sample in the furnace. (b) Avrami rate constants (*k*_A_) and parameters (*n*_A_) for CO_2_ adsorption by m-2-m–Mg_2_(dobpdc) from a 15% stream at temperatures ranging from 55 to 95 °C.

Note that in both models, the constant partial pressure of CO_2_ (*P*/*P*_0_ = 0.15) is embedded in the rate constant *k*_1_ or *k*_A_, respectively, and that *n*_A_ in the Avrami model is also likely a function of *P*/*P*_0_. As expected, a pseudo-first order model using rate constant *k*_1_ (eqn (1)) failed to capture the induction period at temperatures near *T*_step_ (Fig. 6a). In contrast, the Avrami model fits the data well, particularly for temperatures just below the step. We attribute the deviation from the model in the first 1.5 min to incomplete gas mixing in the furnace and to pre-step chemisorption, as discussed above. This model incorporates the Avrami parameter, *n*_A_, as well as the Avrami rate constant, *k*_A_, to produce sigmoidal kinetics profiles, with larger *n*_A_ values leading to longer induction periods. For example, *n*_A_ = 2 corresponds to the sigmoidal kinetics profile of the growth of a one-dimensional crystal.^44,45^

A plot of the Avrami rate constant *k*_A_*vs. T*, which is useful for visualizing trends in these data, is presented in Fig. 6b. This plot indicates that the Avrami rate constant becomes progressively larger with decreasing temperature, reflecting the inverse Arrhenius behavior of m-2-m–Mg_2_(dobpdc) (Fig. 6b). At temperatures below 70 °C, the slope of *k*_A_*vs. T* substantially decreases, possibly due to the intrinsic properties of the material or to instrumentation limitations associated with the high rate of adsorption at these colder temperatures. Unexpectedly, *k*_A_*vs. T* follows a linear trend from 70 to 95 °C, inconsistent with standard Arrhenius behavior (see “Inverse Arrhenius Behavior” below). Notably, the *x*-intercept of this plot (*k*_A_ = 0) should correspond to *T*_step_; indeed, the *x*-intercept of a linear fit to the high-temperature data (70–95 °C) of 100 °C is close to the step temperature determined by cooling a sample of m-2-m–Mg_2_(dobpdc) under 15% CO_2_ (102 °C; see Fig. S8 and Table S2†). In addition, the parameter *n*_A_ progressively decreases from an initial value of 1.7 at 95 °C to a value close to 1 at the lowest temperature of 55 °C, reflecting the longer induction periods near *T*_step_. Therefore, the parameters from these Avrami fits successfully reflect the experimental observations of slower CO_2_ adsorption kinetics (smaller *k*_A_) and longer induction periods (larger *n*_A_) at high temperatures close to the step temperature in m-2-m–Mg_2_(dobpdc). The smaller values of *n*_A_ at lower temperatures reflect shorter induction periods and demonstrate an advantage of maintaining a buffer between the adsorption temperature and the step temperature.

### Inverse Arrhenius behavior

**Fig. 7:**
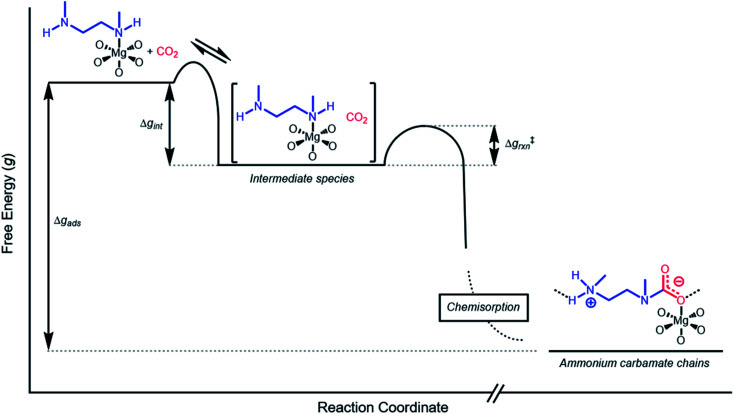
Proposed reaction coordinate diagram for CO_2_ adsorption in m-2-m–Mg_2_(dobpdc) below *T*_step_. The inverse Arrhenius behavior suggests that CO_2_ initially adsorbs in a reversible physisorption or weak chemisorption step, forming an intermediate species that is lower in energy than the starting materials (with energy difference Δ*g*_int_). The intermediate species is then converted to a stable CO_2_ chemisorbed product in a highly exothermic step with activation energy Δ*g*^‡^_rxn_. The inverse Arrhenius behavior suggests that Δ*g*_int_ is larger in magnitude than Δ*g*^‡^_rxn_. We note that ammonium carbamate chains are known to be the final product of CO_2_ chemisorption in m-2-m–Mg_2_(dobpdc), but the exact mechanism and rate-determining step leading to these chains remain unclear.

The inverse Arrhenius behavior observed here can be rationalized using a reaction coordinate diagram derived from several previous investigations of reactions with apparent negative activation energies (Fig. 7).^40,46–49^ In general, inverse Arrhenius behavior requires the reversible formation of an intermediate species with equilibrium constant *K*_eq_ that (i) is lower in energy than the reactant(s), with energy difference Δ*g*_int_; and (ii) proceeds to product formation through a transition state, with kinetic barrier Δ*g*^‡^_rxn_, that is also lower in free energy than the reactant(s), such that |Δ*g*^‡^_rxn_| < |Δ*g*_int_| (see Fig. 7). In addition, the product formation must be an effectively irreversible process, with negligible conversion back to the intermediate species under the reaction conditions. This scenario leads to inverse Arrhenius behavior because the rate of product formation is dependent on the concentration of the intermediate species. Critically, for an adsorption process, this key intermediate species is thermodynamically disfavored at higher temperatures due to the greater entropic penalty associated with removing CO_2_ from the gas phase. In other words, increasing the temperature drives the intermediate species back toward the reactant(s) more than it promotes overcoming the barrier to form the product. In m-2-m–Mg_2_(dobpdc), reversible adsorption of CO_2_ to form a labile intermediate, such as a physisorbed or weakly chemisorbed species, likely precedes an exothermic rate-determining chemisorption step. The final product of chemisorption is an ammonium carbamate chain,^26^ which forms with a thermodynamic free energy change of Δ*g*_ads_. However, C–N bond formation, proton transfer, and metal–oxygen bond formation must all occur between the starting material and the final ammonium carbamate chains; the number of steps in this reaction pathway and the rate-determining step remain points of ongoing investigation. Nevertheless, the temperature dependence of the kinetics of CO_2_ adsorption in m-2-m–Mg_2_(dobpdc) is consistent with an overall mechanistic model involving the reversible formation of an entropically disfavored, CO_2_-bound intermediate followed by a rate-determining chemisorption step. A similar model can be invoked to explain the observed inverse Arrhenius behavior of CO_2_ adsorption in other amine-functionalized adsorbents.^11,37^

**Fig. 8:**
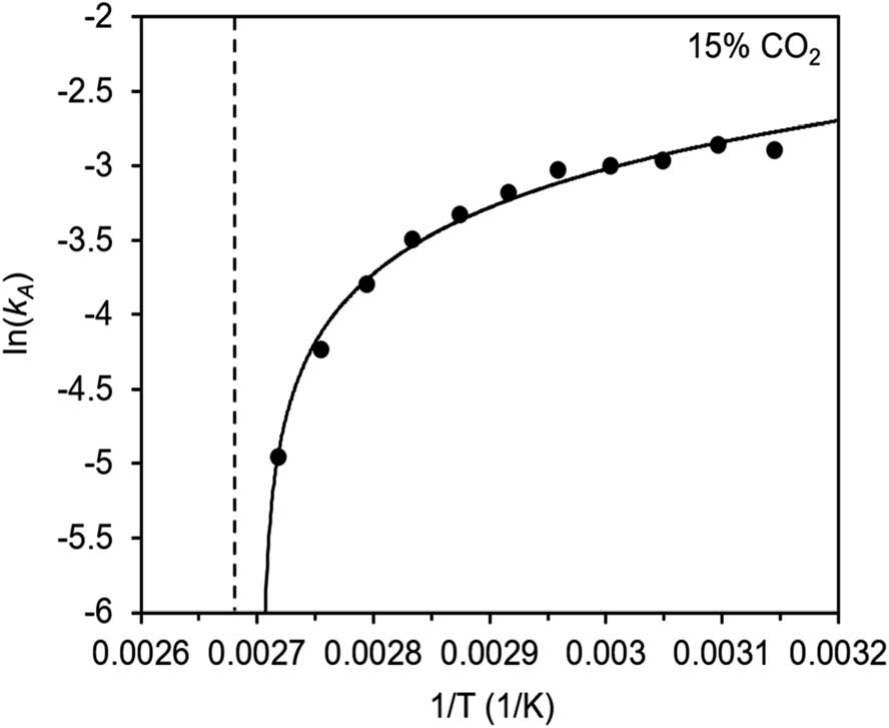
Arrhenius plot for CO_2_ adsorption from a 15% CO_2_ in N_2_ stream by m-2-m–Mg_2_(dobpdc). The dotted line indicates the step temperature under 15% CO_2_. The curved solid line represents a logarithmic fit to the data: *y* = 2.05 + 0.624 ln(*x* − 0.0027). Root mean square error (RMSE): 0.063. Range of the dependent variable: 2.198 (RMSE/range) × 100% = 2.9%.

To further understand the behavior of m-2-m–Mg_2_(dobpdc), we constructed an Arrhenius plot using the Avrami rate constants given in Fig. 6b. As expected, ln(*k*_A_) increases with 1/*T* (Fig. 8), and the sharp curvature occurring near 1/*T*_step_ is consistent with the unusually linear behavior of *k*_A_*vs. T* near *T*_step_ (Fig. 6b). The data can be fit well using a logarithmic function with a vertical asymptote corresponding to ∼97 °C, close to the *T*_step_ of 102 °C (see Fig. S8†). The steeper slope of the Arrhenius plot near 1/*T*_step_ indicates that the temperature dependence of the rate of adsorption in m-2-m–Mg_2_(dobpdc) is greatest close to *T*_step_.

While the reaction coordinate diagram in Fig. 7 accounts for the inverse Arrhenius behavior of CO_2_ adsorption in m-2-m–Mg_2_(dobpdc), it does not necessarily predict the extreme curvature of the Arrhenius plot near 1/*T*_step_. Because cooperative CO_2_ adsorption does not occur above *T*_step_, the Arrhenius plot for the cooperative adsorption process should deviate to −∞ upon approaching *T*_step_ from low to high temperature. Discontinuities have previously been observed in linear Arrhenius plots when a phase change occurs at a specific temperature in an enzyme.^50^ However, the curvature exhibited in the Arrhenius plot of m-2-m–Mg_2_(dobpdc) is unusual and suggests a progressive decrease in the adsorption kinetics as *T* → *T*_step_. The curved Arrhenius plot can potentially be explained by considering the entropy change in the reaction coordinate diagram in Fig. 7, as has been previously described.^51^ Moving from left to right along the reaction coordinate in Fig. 7 corresponds to a significant decrease in degrees of freedom as gaseous CO_2_ is immobilized and the initially dynamic diamines are locked into ammonium carbamate chains. As the temperature increases, the entropic penalty for immobilizing CO_2_ increases and ultimately outweighs the enthalpic favorability of adsorption when *T* exceeds *T*_step_. Accordingly, the kinetic barrier of the rate-determining chemisorption step should become increasingly large at higher temperatures, consequently slowing the reaction kinetics. Therefore, increasing the temperature towards *T*_step_ imposes two compounding deleterious entropic effects on the rate of adsorption in m-2-m–Mg_2_(dobpdc): it thermodynamically disfavors the formation of the intermediate species (as discussed above), and it increases the magnitude of the rate-limiting kinetic barrier. Together, these effects can potentially explain the observed progressively slower adsorption kinetics and resulting curved Arrhenius plot as *T* approaches *T*_step_. Determining the identity of the intermediate species and elucidating the mechanism of the rate-limiting chemisorption process remain active areas of investigation.

### A structure–property kinetics relationship using a panel of ethylenediamine analogues

**Fig. 9:**
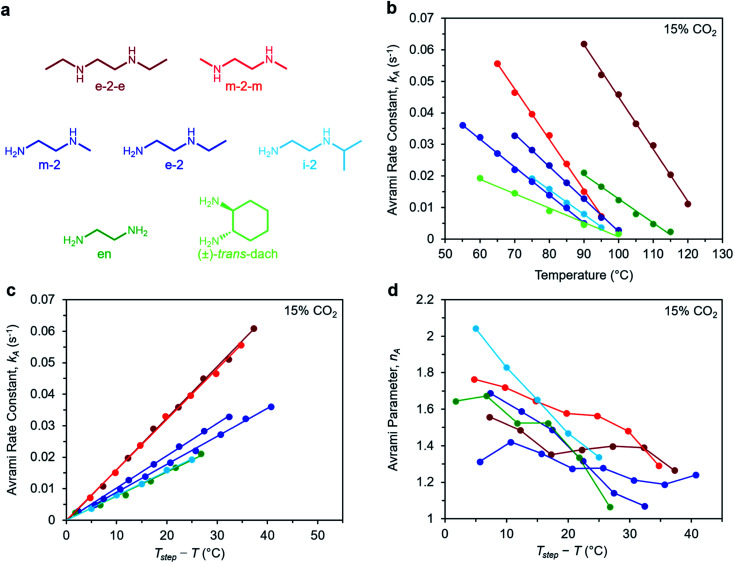
Rate constants and Avrami parameters for 15% CO_2_ adsorption by diamine-appended Mg_2_(dobpdc) variants. (a) Structures of diamines evaluated. The color scheme corresponds to the plots in (b)–(d). (b) Plot of Avrami rate constant (*k*_A_) *vs. T* for diamine-appended variants. Colored lines depict linear fits to the data. Linear regression data are shown in Fig. S26.† (c) Plot of *k*_A_ data *vs. T*_step_ − *T*, where *T* is the experimental adsorption temperature and *T*_step_ is the adsorption step temperature (*T*_step_ values correspond to the *x*-intercepts of the linear fits in (b)). (d) Plot of Avrami parameter (*n*_A_) *vs. T* for diamine-appended variants of Mg_2_(dobpdc). The *x* axis units are the same as in (c). Note that panels (c) and (d) do not contain data for (±)-*trans*-dach–Mg_2_(dobpdc) because it was not possible to approximate *T*_step_ based on the linear fit shown in (b); see Fig. S19–20.†

To determine the generalizability of these characteristics for m-2-m–Mg_2_(dobpdc) to other diamine-appended frameworks, we evaluated the kinetics of CO_2_ adsorption from a gas stream of 15% CO_2_ in N_2_ by a panel of Mg_2_(dobpdc) variants appended with structurally-diverse alkylethylenediamines. Notably, frameworks with 1°,3° alkylethylenediamines^26^ displayed minimal adsorption at temperatures ≥40 °C (Fig. S12†) and thus were not investigated further. In contrast, rate constants could be determined for frameworks appended with 1°,1°; 1°,2°; and 2°,2° amines (see Fig. 9). We note that the diamines *N*-isopropylethylenediamine (i-2) and *N*,*N*′-diethylethylenediamine (e-2-e) exhibit two-step adsorption behavior,^28^ and thus their kinetic profiles are complex at temperatures at which both steps are operative. As a result, Fig. 9 depicts only the temperatures under which the less thermodynamically favorable step is not operative for these two diamine-appended frameworks.

A similar overall sigmoidal kinetic profile was observed for all ethylenediamine variants in Fig. 9, consistent with our previous findings that these materials capture CO_2_ by the same mechanism.^26,52^ In each case, *k*_A_ increases with decreasing temperature, indicative of inverse Arrhenius behavior, as observed for m-2-m–Mg_2_(dobpdc) (Fig. S13–S18†). Furthermore, all of the variants exhibit linear plots of *k*_A_*vs. T* (Fig. 9b) and curved Arrhenius plots that could be fit by logarithmic functions (Fig. S13–S18†), suggesting that the complex behavior characterized for m-2-m–Mg_2_(dobpdc) represents a general feature of these materials. In Fig. 9b, the linear fits to the *k*_A_*vs. T* data yield different *x* intercepts for each diamine-appended framework, reflecting their different adsorption step temperatures. The *x*-intercepts all match closely to step temperatures determined by cooling the frameworks under 15% CO_2_ (Fig. S8–S11 and Table S2†), except in the case of (±)-*trans*-dach–Mg_2_(dobpdc), for which the *k*_A_*vs. T* plot deviates from linearity at high temperatures (Fig. S20†).

The differences in adsorption step temperatures complicate comparisons between materials because the free energy change associated with CO_2_ adsorption (Δ*g*_ads_) is different for each material at a constant temperature. To account for this difference, we also plotted *k*_A_*vs. T*_step_ − *T* to scale the *x*-intercepts of the linear fits to zero (Fig. 9c). Note that we do not consider *T*_step_ − *T* to be a physically meaningful metric of chemical potential—rather, this plot is only intended to help visualize differences among diamine-appended frameworks. Importantly, the linear fits to the data in the corresponding plots all exhibit distinct slopes, reflecting variability in the extent to which decreasing the temperature below *T*_step_ increases the CO_2_ adsorption kinetics. For practical applications, a steep slope in Fig. 9c is desirable to enable fast adsorption kinetics even at temperatures just below the step. In the plot of *n*_A_*vs. T*_step_ − *T* (Fig. 9d), *n*_A_ generally becomes smaller with decreasing temperature. Most of the materials exhibit values of *n*_A_ between 1.3 and 1.8 at temperatures close to the step, decreasing to ∼1 at the lowest temperatures. Materials with smaller values of *n*_A_ show less pronounced induction periods and are therefore preferable for implementation in a process.

Interestingly, the steepest slopes were observed for frameworks appended with the 2°,2° diamines m-2-m and e-2-e.^24–26^ These variants are notable for having weak metal–amine bonds, which serve to increase |Δ*h*_ads_| for CO_2_ and facilitate CO_2_ insertion, thus likely contributing to their fast adsorption kinetics.^26^ Despite these desirable kinetics properties, the weak metal–amine bonds lead to diamine volatilization under humid conditions, particularly at the high temperatures necessary for desorption under a pure CO_2_ stream.^53,54^ The second-fastest kinetics were found for variants of Mg_2_(dobpdc) functionalized with the 1°,2° diamines *N*-methylethylenediamine (m-2), *N*-ethylethylenediamine (e-2), and i-2.^26,28,55^ We previously determined that the primary amine preferentially binds to the metal site and reacts with CO_2_, which may account for the slower adsorption kinetics in these materials compared to those functionalized with 2°,2° diamines.^26^ However, this effect also bestows 1°,2° diamine-appended variants with enhanced stability toward diamine loss.^26,28^ The compounds with the least sterically-encumbered 1°,2° diamines exhibit slightly faster kinetics, as indicated by steeper slopes in the plots of *k*_A_*vs. T* (Fig. 9c). Overall, these results suggest a tradeoff between stability and adsorption kinetics for frameworks appended with 1°,2° diamines compared to those bearing 2°,2° diamines.

Finally, we studied the behavior of Mg_2_(dobpdc) variants appended with the 1°,1° diamines ethylenediamine (en) and (±)-*trans*-diaminocyclohexane (dach),^26,29,31,56^ which have previously been shown to exhibit significant adsorption/desorption hysteresis and unit cell contraction upon CO_2_ adsorption.^56^ Interestingly, the CO_2_ adsorption kinetics of en–Mg_2_(dobpdc) are extremely similar to those of i-2–Mg_2_(dobpdc)—the 1°,2° variant with the slowest CO_2_ adsorption kinetics—while (±)-*trans*-dach–Mg_2_(dobpdc) exhibits much slower kinetics. In addition, (±)-*trans*-dach–Mg_2_(dobpdc) displays an unusually high *n*_A_ of ∼3 at temperatures close to the step, corresponding to a very long induction period (Fig. S19–S21†). As with the other diamines, the adsorption kinetics in (±)-*trans*-dach–Mg_2_(dobpdc) become much faster and the induction periods become much shorter at lower temperatures (*n*_A_ = 1.3 at 60 °C; see Fig. S21†), and use of a fast flow rate (100 mL min^−1^) also shortens the induction period for this material (Fig. S22†). One possible explanation for the large *n*_A_ value and slower overall kinetics of (±)-*trans*-dach–Mg_2_(dobpdc) is the unit cell contraction that occurs upon CO_2_ adsorption in conjunction with crystallographically characterized ion-pairing interactions between neighboring ammonium carbamate chains in the *ab* plane.^56^ Intriguingly, *n*_A_ = 3 for the Avrami model corresponds to the growth of a crystal in two dimensions, whereas *n*_A_ = 2 corresponds to growth in one dimension.^44,45^ Accordingly, cooperative CO_2_ adsorption in (±)-*trans*-dach–Mg_2_(dobpdc) may be akin to two-dimensional sheet growth, whereas cooperative CO_2_ capture by the other variants may be more akin to one-dimensional chain growth. Elucidating the potential correlation between *ab* plane contraction and the CO_2_ adsorption kinetics for 1°,1° diamine–Mg_2_(dobpdc) variants remains a subject of investigation.

### Adsorption kinetics of 2,2-dimethyl-1,3-diaminopropane–Mg_2_(dobpdc)

In addition to ethylenediamines, Mg_2_(dobpdc) has also been appended with diaminopropanes to yield cooperative adsorbents.^27^ For example, dmpn–Mg_2_(dobpdc) (dmpn = 2,2-dimethyl-1,3-diaminopropane) has a favorable adsorption step temperature for CO_2_ capture from coal flue gas and maintains a high working capacity over 1000 cycles under humid conditions.^27^ Notably, the complex CO_2_ chemisorption mechanism in dmpn–Mg_2_(dobpdc) ultimately leads to the formation of carbamic acids interacting with ammonium carbamates, and this material exhibits a mixture of ∼4 chemisorbed species in the early stages of CO_2_ adsorption.^52^ Given this complexity and the promising attributes of dmpn–Mg_2_(dobpdc) for implementation in practical processes, we sought to investigate its adsorption kinetics from a gas stream of 15% CO_2_ in N_2_.

**Fig. 10:**
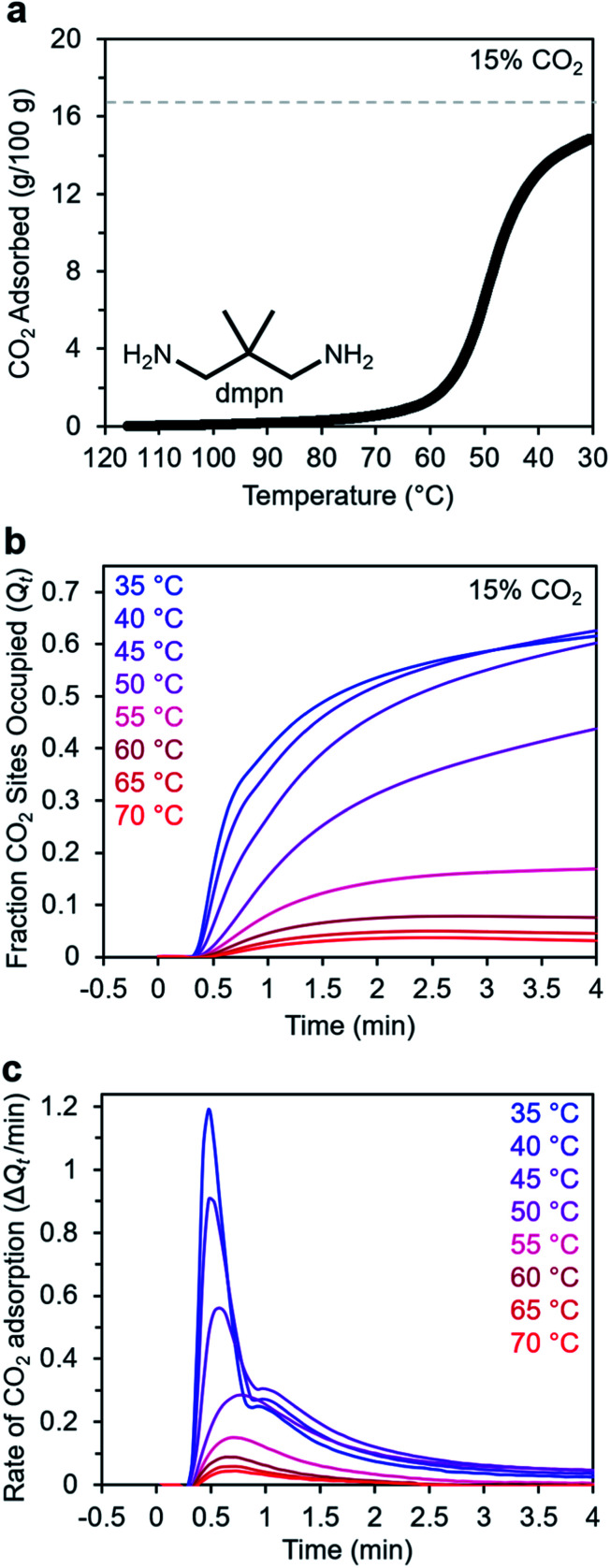
Kinetics of CO_2_ adsorption in dmpn–Mg_2_(dobpdc). (a) 15% CO_2_ adsorption isobar for dmpn–Mg_2_(dobpdc) following activation at 120 °C for 20 min. The gray dashed line represents the adsorption capacity corresponding 1 CO_2_ per diamine (16.8 g/100 g). (b) Adsorption from a 15% CO_2_ stream *vs.* time for dmpn–Mg_2_(dobpdc). Sample activation: 100 °C for 5 min under N_2_. (c) Rate of CO_2_ adsorption *vs.* time corresponding to the data shown in (b). A 19-s delay is observed prior to the sample mass increasing, corresponding to the time required for CO_2_ to reach the sample in the furnace.

Consistent with previous findings, the 15% CO_2_ adsorption isobar for this material exhibits a step around 60 °C that is more broad than those of other diamine–appended Mg_2_(dobpdc) variants, reflecting its complex adsorption mechanism (Fig. 10a).^27,52^ Similarly, the kinetics profile of dmpn–Mg_2_(dobpdc) is distinct from that of the other diamine-appended variants (Fig. 10b and c), with no induction period observed even at temperatures close to the adsorption step. At all temperatures below 55 °C, dmpn–Mg_2_(dobpdc) exhibits a very rapid initial uptake, followed by slower uptake until reaching equilibrium. This unusual curve shape results in Avrami fits with small *n*_A_ (∼0.4–0.7) and *k*_A_ (∼0.003–0.006 s^−1^) values (Fig. S23†). The fastest initial uptake in dmpn–Mg_2_(dobpdc) occurs at 35 °C—the lowest temperature investigated—but only up to an occupancy of ∼0.6, after which the kinetics slow (Fig. S24†). We note that this slower adsorption at low temperatures and high occupancies was not observed in a comparable temperature range for the other diamine-appended variants, but a similar effect was reported previously in “molecular basket” polyamine-appended mesoporous silicas.^10^ Overall, the lack of induction period for dmpn–Mg_2_(dobpdc) is promising for implementation in a CO_2_ capture process.

### Desorption kinetics

Desorption kinetics are also a critical consideration for the practical use of an adsorbent. In a typical fixed-bed process, adsorption and desorption stages are operated simultaneously using a minimum of two beds. As a consequence, fast desorption kinetics are desirable to minimize cycle times. In a simulation of the desorption conditions typical of a temperature swing adsorption process, we previously demonstrated that CO_2_ can be desorbed from diamine-appended Mg_2_(dobpdc) under a humidified stream of pure CO_2_ within less than 10 min, as limited by the heating rate of the TGA furnace.^27,28^ Alternatively, the introduction of dry N_2_ as a purge gas can be used to simulate the application of a concentration- or vacuum-swing process. Because diamine-appended Mg_2_(dobpdc) variants can maintain high CO_2_ capacities under pure CO_2_ even at relatively high temperatures, these materials can be heated under CO_2_ to a temperature of interest and allowed to equilibrate, after which the gas stream can be switched to dry N_2_ and the decrease in mass can be monitored.

**Fig. 11:**
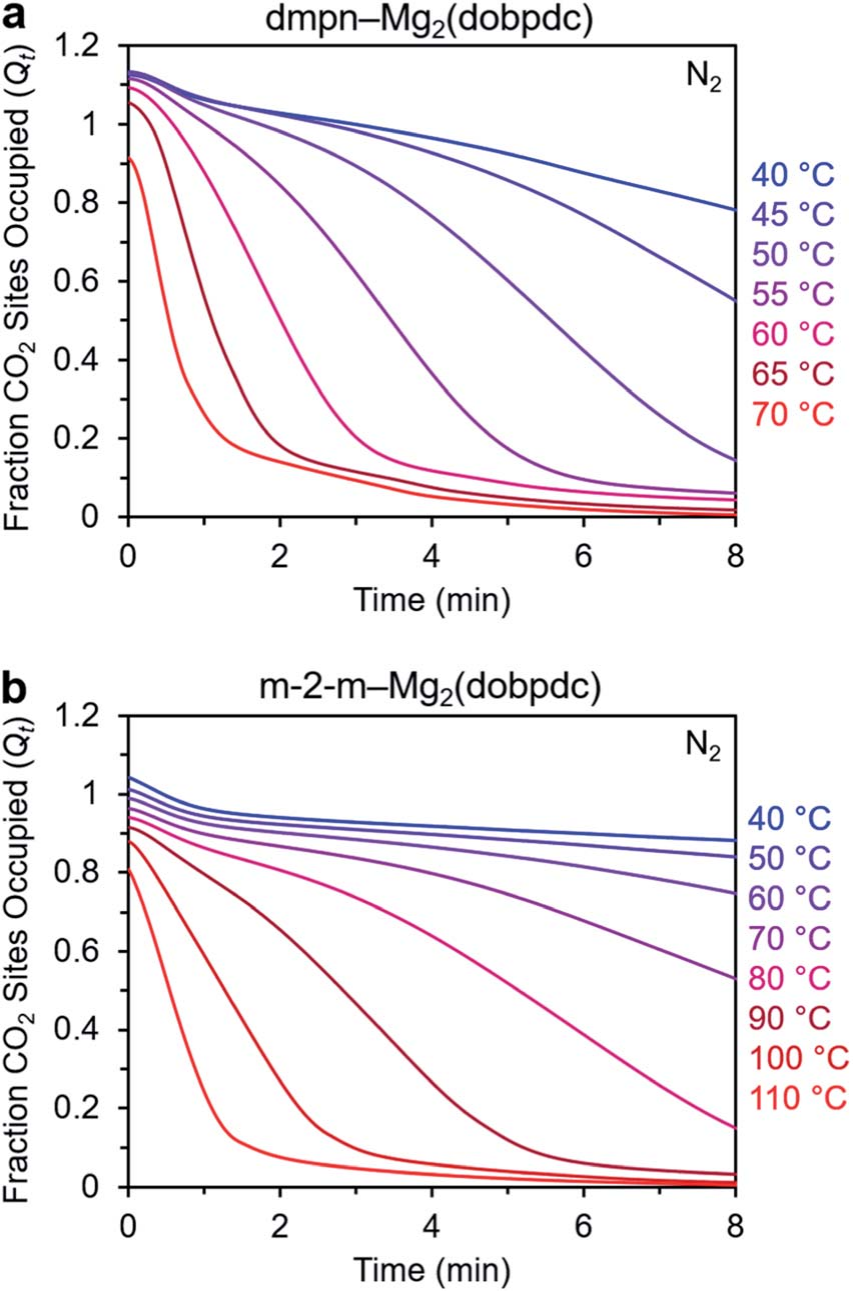
Desorption of CO_2_ under dry N_2_ from (a) dmpn–Mg_2_(dobpdc) and (b) m-2-m–Mg_2_(dobpdc). In each experiment, the sample was allowed to equilibrate under pure CO_2_ at the indicated temperature, after which the gas stream was switched to N_2_. In these plots, data are normalized such that *t* = 0 corresponds to the data point immediately before mass decrease was observed due to CO_2_ desorption (*i.e.*, *t* = 0 is after the 19 s delay prior to N_2_ reaching the sample in the furnace).

We utilized this dry N_2_ desorption assay to compare the desorption kinetics of dmpn–Mg_2_(dobpdc) and m-2-m–Mg_2_(dobpdc) (Fig. 11). At the highest temperature investigated for each material (70 and 110 °C for dmpn and m-2-m, respectively), nearly all of the CO_2_ was desorbed within 1.5 min, and decreasing the desorption temperature progressively decreased the rate of desorption. For each adsorbent, the initial *Q*_*t*_ values varied with temperature, consistent with the isobaric adsorption profiles, and the larger *Q*_*t*_ values at colder temperatures can be attributed to CO_2_ physisorption. Overall, desorption from dmpn–Mg_2_(dobpdc) occurs in a lower temperature range compared to desorption from m-2-m–Mg_2_(dobpdc), reflecting the lower desorption step temperature for the dmpn variant of 93 °C ^27^ compared to 134 °C for m-2-m,^26^ as determined from dry 100% CO_2_ desorption isobars collected using a ramp rate of 1 °C min^−1^.

As shown in Fig. S25,† the desorption kinetics for dmpn–Mg_2_(dobpdc) and m-2-m–Mg_2_(dobpdc) fit well to the Avrami model across multiple temperatures. For both materials, desorption is initially slow, but is accelerated after some CO_2_ has been desorbed. Interestingly, the desorption kinetics behavior of dmpn–Mg_2_(dobpdc) differs from its adsorption kinetics behavior. While this material exhibits no induction period for adsorption (*n*_A_ ∼0.5), a substantial induction period was observed for desorption (*n*_A_ ∼2 for many of the conditions tested; see Fig. S25†). These dissimilar kinetics profiles may result from dmpn–Mg_2_(dobpdc) exhibiting a mixture of approximately four chemisorbed species in the early stages of CO_2_ adsorption, while a 1 : 1 mixture of ammonium carbamates:carbamic acids is formed following saturation with CO_2_ and long equilibration times.^52^

## Conclusions

The foregoing results describe a systematic investigation into the CO_2_ adsorption kinetics of diamine-appended variants of the metal–organic framework Mg_2_(dobpdc) using a TGA-based assay. Sigmoidal kinetics profiles were observed for all of the materials that exclusively form ammonium carbamate chains upon CO_2_ adsorption, which we attribute to their cooperative adsorption mechanism. While these adsorbents exhibit long induction periods at temperatures near the adsorption step, the kinetics of adsorption can be accelerated and the length of the induction period minimized by decreasing the adsorption temperature. Additionally, the diamine structure can be optimized to yield adsorption thermodynamics and kinetics that are most favorable for a target gas separation involving CO_2_. Finally, no induction period was observed for the material dmpn–Mg_2_(dobpdc), which adsorbs CO_2_*via* a mixed ammonium carbamate/carbamic acid mechanism. Thus, accessing new chemisorption products within the diamine-appended materials may give rise to advantageous changes in kinetics as well as thermodynamics. Overall, this study provides key guidelines for utilizing diamine-appended variants of Mg_2_(dobpdc) in CO_2_ separations and should prove more broadly useful for the design of new chemisorptive adsorbents for carbon capture applications.

## Conflicts of interest

The authors declare the following competing financial interest: J. R. L. has a financial interest in Mosaic Materials, Inc., a start-up company working to commercialize metal–organic frameworks for gas separations, including CO_2_ capture applications. The University of California, Berkeley has applied for a patent on some of the materials discussed herein, on which J. R. L., P. J. M., and R. L. S. are listed as inventors.

## Supplementary Material

SC-011-D0SC01087A-s001

## References

[cit1] Rogelj J.; Shindell D.; Jiang K.; Fifita S.; Forster P.; Ginzburg V.; Handa C.; Kheshgi H., Kobayashi S. and Kriegler E., Mitigation Pathways Compatible with 1.5 °C in the Context of Sustainable Development, in Global Warming of 1.5°C. An IPCC Special Report on the impacts of global warming of 1.5°C above pre-industrial levels and related global greenhouse gas emission pathways, in the context of strengthening the global response to the threat of climate change, sustainable development, and efforts to eradicate poverty, 2018

[cit2] Jackson R. B., Le Quéré C., Andrew R. M., Canadell J. G., Peters G. P., Roy J., Wu L. (2017). Warning Signs for Stabilizing Global CO_2_ Emissions. Environ. Res. Lett..

[cit3] Boot-Handford M. E., Abanades J. C., Anthony E. J., Blunt M. J., Brandani S., Mac Dowell N., Fernandez J. R., Ferrari M.-C., Gross R., Hallett J. P. (2014). *et al.*, Carbon Capture and Storage Update. Energy Environ. Sci..

[cit4] Yang R. T., Adsorber Dynamics: Bed Profiles and Breakthrough Curves, in Gas Separation by Adsorption Processes, Butterworth-Heinemann, Boston, 1987, ch. 5, pp. 141–200

[cit5] Bhown A. S., Freeman B. C. (2011). Analysis and Status of Post-Combustion Carbon Dioxide Capture Technologies. Environ. Sci. Technol..

[cit6] Ramezan M., Skone T. J., Nsakala N. Y., Liljedahl G. N., Gearhart L. E., Hestermann R. and Rederstorff B., Carbon Dioxide Capture from Existing Coal-Fired Power Plants, Natl. Energy Technol. Lab. DOENETL Rep., 2007, no. 401/110907

[cit7] Mason J. A., McDonald T. M., Bae T.-H., Bachman J. E., Sumida K., Dutton J. J., Kaye S. S., Long J. R. (2015). Application of a High-Throughput Analyzer in Evaluating Solid Adsorbents for Post-Combustion Carbon Capture via Multicomponent Adsorption of CO_2_, N_2_, and H_2_O. J. Am. Chem. Soc..

[cit8] Leal O., Bolívar C., Ovalles C., García J. J., Espidel Y. (1995). Reversible Adsorption of Carbon Dioxide on Amine Surface-Bonded Silica Gel. Inorg. Chim. Acta.

[cit9] Mello M. R., Phanon D., Silveira G. Q., Llewellyn P. L., Ronconi C. M. (2011). Amine-Modified MCM-41 Mesoporous Silica for Carbon Dioxide Capture. Microporous Mesoporous Mater..

[cit10] Ma X., Wang X., Song C. (2009). “Molecular Basket” Sorbents for Separation of CO_2_ and H_2_S from Various Gas Streams. J. Am. Chem. Soc..

[cit11] Monazam E. R., Shadle L. J., Miller D. C., Pennline H. W., Fauth D. J., Hoffman J. S., Gray M. L. (2013). Equilibrium and Kinetics Analysis of Carbon Dioxide Capture Using Immobilized Amine on a Mesoporous Silica. AIChE J..

[cit12] Yang Y., Chuah C. Y., Bae T.-H. (2019). Polyamine-Appended Porous Organic Polymers for Efficient Post-Combustion CO_2_ Capture. Chem. Eng. J..

[cit13] Lu W., Sculley J. P., Yuan D., Krishna R., Wei Z., Zhou H.-C. (2012). Polyamine-Tethered Porous Polymer Networks for Carbon Dioxide Capture from Flue Gas. Angew. Chem..

[cit14] Kim C., Cho H. S., Chang S., Cho S. J., Choi M. (2016). An Ethylenediamine-Grafted Y Zeolite: A Highly Regenerable Carbon Dioxide Adsorbent via Temperature Swing Adsorption without Urea Formation. Energy Environ. Sci..

[cit15] Su F., Lu C., Kuo S.-C., Zeng W. (2010). Adsorption of CO_2_ on Amine-Functionalized Y-Type Zeolites. Energy
Fuels.

[cit16] Demessence A., D'Alessandro D. M., Foo M. L., Long J. R. (2009). Strong CO_2_ Binding in a Water-Stable, Triazolate-Bridged Metal–Organic Framework Functionalized with Ethylenediamine. J. Am. Chem. Soc..

[cit17] McDonald T. M., D'Alessandro D. M., Krishna R., Long J. R. (2011). Enhanced Carbon Dioxide Capture upon Incorporation of *N*,*N*′-Dimethylethylenediamine in the Metal–Organic Framework CuBTTri. Chem. Sci..

[cit18] Choi S., Watanabe T., Bae T.-H., Sholl D. S., Jones C. W. (2012). Modification of the Mg/DOBDC MOF with Amines to Enhance CO_2_ Adsorption from Ultradilute Gases. J. Phys. Chem. Lett..

[cit19] Liao P.-Q., Chen X.-W., Liu S.-Y., Li X.-Y., Xu Y.-T., Tang M., Rui Z., Ji H., Zhang J.-P., Chen X.-M. (2016). Putting an Ultrahigh Concentration of Amine Groups into a Metal–Organic Framework for CO_2_ Capture at Low Pressures. Chem. Sci..

[cit20] Darunte L. A., Oetomo A. D., Walton K. S., Sholl D. S., Jones C. W. (2016). Direct Air Capture of CO_2_ Using Amine Functionalized MIL-101(Cr). ACS Sustainable Chem. Eng..

[cit21] Wang Z., Cohen S. M. (2007). Postsynthetic Covalent Modification of a Neutral Metal−Organic Framework. J. Am. Chem. Soc..

[cit22] Jiang H.-L., Feng D., Liu T.-F., Li J.-R., Zhou H.-C. (2012). Pore Surface Engineering with Controlled Loadings of Functional Groups via Click Chemistry in Highly Stable Metal–Organic Frameworks. J. Am. Chem. Soc..

[cit23] Liu J., Wang Y., Benin A. I., Jakubczak P., Willis R. R., LeVan M. D. (2010). CO_2_/H_2_O Adsorption Equilibrium and Rates on Metal−Organic Frameworks: HKUST-1 and Ni/DOBDC. Langmuir.

[cit24] McDonald T. M., Lee W. R., Mason J. A., Wiers B. M., Hong C. S., Long J. R. (2012). Capture of Carbon Dioxide from Air and Flue Gas in the Alkylamine-Appended Metal–Organic Framework mmen-Mg_2_(dobpdc). J. Am. Chem. Soc..

[cit25] McDonald T. M., Mason J. A., Kong X., Bloch E. D., Gygi D., Dani A., Crocella V., Giordanino F., Odoh S. O., Drisdell W. S. (2015). *et al.*, Cooperative Insertion of CO_2_ in Diamine-Appended Metal-Organic Frameworks. Nature.

[cit26] Siegelman R. L., McDonald T. M., Gonzalez M. I., Martell J. D., Milner P. J., Mason J. A., Berger A. H., Bhown A. S., Long J. R. (2017). Controlling Cooperative CO_2_ Adsorption in Diamine-Appended Mg_2_(dobpdc) Metal–Organic Frameworks. J. Am. Chem. Soc..

[cit27] Milner P. J., Siegelman R. L., Forse A. C., Gonzalez M. I., Runčevski T., Martell J. D., Reimer J. A., Long J. R. (2017). A Diaminopropane-Appended Metal–Organic Framework Enabling Efficient CO_2_ Capture from Coal Flue Gas via a Mixed Adsorption Mechanism. J. Am. Chem. Soc..

[cit28] Milner P. J., Martell J. D., Siegelman R. L., Gygi D., Weston S. C., Long J. R. (2018). Overcoming Double-Step CO_2_ Adsorption and Minimizing Water Co-Adsorption in Bulky Diamine-Appended Variants of Mg_2_(dobpdc). Chem. Sci..

[cit29] Lee W. R., Hwang S. Y., Ryu D. W., Lim K. S., Han S. S., Moon D., Choi J., Hong C. S. (2014). Diamine-Functionalized Metal–Organic Framework: Exceptionally High CO_2_ Capacities from Ambient Air and Flue Gas, Ultrafast CO_2_ Uptake Rate, and Adsorption Mechanism. Energy Environ. Sci..

[cit30] Lee W. R., Jo H., Yang L.-M., Lee H., Ryu D. W., Lim K. S., Song J. H., Min D. Y., Han S. S., Seo J. G. (2015). *et al.*, Exceptional CO_2_ Working Capacity in a Heterodiamine-Grafted Metal–Organic Framework. Chem. Sci..

[cit31] Jo H., Lee W. R., Kim N. W., Jung H., Lim K. S., Kim J. E., Kang D. W., Lee H., Hiremath V., Seo J. G. (2017). *et al.*, Fine-Tuning of the Carbon Dioxide Capture Capability of Diamine-Grafted Metal–Organic Framework Adsorbents Through Amine Functionalization. ChemSusChem.

[cit32] Heydari-Gorji A., Sayari A. (2011). CO_2_ Capture on Polyethylenimine-Impregnated Hydrophobic Mesoporous Silica: Experimental and Kinetic Modeling. Chem. Eng. J..

[cit33] Ebner A. D., Gray M. L., Chisholm N. G., Black Q. T., Mumford D. D., Nicholson M. A., Ritter J. A. (2011). Suitability of a Solid Amine Sorbent for CO_2_ Capture by Pressure Swing Adsorption. Ind. Eng. Chem. Res..

[cit34] Monazam E. R., Shadle L. J., Siriwardane R. (2011). Equilibrium and Absorption Kinetics of Carbon Dioxide by Solid Supported Amine Sorbent. AIChE J..

[cit35] Wang J., Stevens L. A., Drage T. C., Wood J. (2012). Preparation and CO_2_ Adsorption of Amine Modified Mg–Al LDH via Exfoliation Route. Chem. Eng. Sci..

[cit36] Granite E. J., Pennline H. W. (2002). Photochemical Removal of Mercury from Flue Gas. Ind. Eng. Chem. Res..

[cit37] Liu Q., Shi J., Zheng S., Tao M., He Y., Shi Y. (2014). Kinetics Studies of CO_2_ Adsorption/Desorption on Amine-Functionalized Multiwalled Carbon Nanotubes. Ind. Eng. Chem. Res..

[cit38] Serna-Guerrero R., Sayari A. (2010). Modeling Adsorption of CO_2_ on Amine-Functionalized Mesoporous Silica. 2: Kinetics and Breakthrough Curves. Chem. Eng. J..

[cit39] Mason J. A., Sumida K., Herm Z. R., Krishna R., Long J. R. (2011). Evaluating Metal–Organic Frameworks for Post-Combustion Carbon Dioxide Capture via Temperature Swing Adsorption. Energy Environ. Sci..

[cit40] Quaranta M., Gehring T., Odell B., Brown J. M., Blackmond D. G. (2010). Unusual Inverse Temperature Dependence on Reaction Rate in the Asymmetric Autocatalytic Alkylation of Pyrimidyl Aldehydes. J. Am. Chem. Soc..

[cit41] Mathew S. P., Klussmann M., Iwamura H., Wells J., David H., Armstrong A., Blackmond D. G. (2006). A Mechanistic Rationalization of Unusual Kinetic Behavior in Proline-Mediated C–O and C–N Bond-Forming Reactions. Chem. Commun..

[cit42] Darunte L. A., Sen T., Bhawanani C., Walton K. S., Sholl D. S., Realff M. J., Jones C. W. (2019). Moving Beyond Adsorption Capacity in Design of Adsorbents for CO_2_ Capture from Ultradilute Feeds: Kinetics of CO_2_ Adsorption in Materials with Stepped Isotherms. Ind. Eng. Chem. Res..

[cit43] Avrami M. (1939). Kinetics of Phase Change. I General Theory. J. Chem. Phys..

[cit44] Ruitenberg G., Petford-Long A. K., Doole R. C. (2002). Determination of the Isothermal Nucleation and Growth Parameters for the Crystallization of Thin Ge_2_Sb_2_Te_5_ Films. J. Appl. Phys..

[cit45] Ranganathan S., Von Heimendahl M. (1981). The Three Activation Energies with Isothermal Transformations: Applications to Metallic Glasses. J. Mater. Sci..

[cit46] Oliveberg M., Tan Y. J., Fersht A. R. (1995). Negative Activation Enthalpies in the Kinetics of Protein Folding. Proc. Natl. Acad. Sci. U. S. A..

[cit47] Moss R. A., Wang L., Zhang M., Skalit C., Krogh-Jespersen K. (2008). Enthalpy versus Entropy in Chlorocarbene/Alkene Addition Reactions. J. Am. Chem. Soc..

[cit48] Houk K. N., Rondan N. G., Mareda J. (1985). Theoretical Studies of Halocarbene Cycloaddition Selectivities: A New Interpretation of Negative Activation Energies and Entropy Control of Selectivity. Tetrahedron.

[cit49] Mozurkewich M., Benson S. W. (1984). Negative Activation Energies and Curved Arrhenius Plots. 1. Theory of Reactions over Potential Wells. J. Phys. Chem..

[cit50] Biosca J. A., Travers F., Barman T. E. (1983). A Jump in an Arrhenius Plot Can Be the Consequence of a Phase Transition: The Binding of ATP to Myosin Subfragment 1. FEBS Lett..

[cit51] Truhlar D. G., Kohen A. (2001). Convex Arrhenius Plots and Their Interpretation. Proc. Natl. Acad. Sci. U. S. A..

[cit52] Forse A. C., Milner P. J., Lee J.-H., Redfearn H. N., Oktawiec J., Siegelman R. L., Martell J. D., Dinakar B., Porter-Zasada L. B., Gonzalez M. I. (2018). Elucidating CO_2_ Chemisorption in Diamine-Appended Metal–Organic Frameworks. J. Am. Chem. Soc..

[cit53] Luz I., Soukri M., Lail M. (2018). Synthesis of Fluidized CO_2_ Sorbents Based on Diamine Coordinated to Metal–Organic Frameworks by Direct Conversion of Metal Oxides Supported on Mesoporous Silica. Chem.–Eur. J..

[cit54] Liang W., Bhatt P. M., Shkurenko A., Adil K., Mouchaham G., Aggarwal H., Mallick A., Jamal A., Belmabkhout Y., Eddaoudi M. (2019). A Tailor-Made Interpenetrated MOF with Exceptional Carbon-Capture Performance from Flue Gas. Chem.

[cit55] Lee W. R., Kim J. E., Lee S. J., Kang M., Kang D. W., Lee H. Y., Hiremath V., Seo J. G., Jin H., Moon D. (2018). *et al.*, Diamine-Functionalization of a Metal–Organic Framework Adsorbent for Superb Carbon Dioxide Adsorption and Desorption Properties. ChemSusChem.

[cit56] Martell J. D., Porter-Zasada L. B., Forse A. C., Siegelman R. L., Gonzalez M. I., Oktawiec J., Runčevski T., Xu J., Srebro-Hooper M., Milner P. J. (2017). *et al.*, Enantioselective Recognition of Ammonium Carbamates in a Chiral Metal–Organic Framework. J. Am. Chem. Soc..

