# Supplementary material for: Kinetics of cooperative CO2 adsorption in diamine-appended variants of the metal–organic framework Mg2(dobpdc)
Source: Chem Sci. 2020 Mar 31;11(25):6457–71. doi: 10.1039/d0sc01087a (PMC8152673; doi:10.1039/d0sc01087a)
Supplement: SC-011-D0SC01087A-s001 [file SC-011-D0SC01087A-s001.pdf]

Electronic Supplementary Material (ESI) for Chemical Science.
This journal is © The Royal Society of Chemistry 2020

Supporting Information for

Kinetics of Cooperative CO_2 Adsorption in Diamine-Appended Variants of the Metal–Organic Framework Mg_2(dobpdc)

Authors: Jeffrey D. Martell,[a,b,e] Phillip J. Milner,[a,b,f] Rebecca L. Siegelman,[b,g] Jeffrey R. Long*,[b,c,d]

[a]These authors contributed equally to this work

[b]Department of Chemistry, University of California, Berkeley, CA 94720, United States

[c]Department of Chemical and Biomolecular Engineering, University of California, Berkeley, CA 94720, United States

[d]Materials Sciences Division, Lawrence Berkeley National Laboratory, Berkeley, CA 94720, United States

[e]Present address: University of Wisconsin–Madison, Department of Chemistry, 1101 University Avenue, Madison, WI 53706, United States

[f]Present address: 328 Baker Laboratory, Cornell University, Ithaca, NY 14853, United States

[g]Present address: DuPont de Nemours, Inc. 200 Powder Mill Rd, Wilmington, DE 19803, United States

*To whom correspondence should be addressed: jrlong@berkeley.edu

EXPERIMENTAL SECTION

General procedures, materials, and reagents. All solvents and diamines were purchased from commercial sources and used as received. The ligand 4,4′-dihydroxy-(1,1′-biphenyl)-3,3′-dicarboxylic acid (H_4dobpdc) was purchased from Hangzhou Trylead Chemical Technology Co.

Synthesis of Mg_2(dobpdc). The compound Mg_2(dobpdc) was synthesized as described previously[1] with slight modifications to the procedure. The ligand H_4dobpdc (9.89 g, 36.1 mmol) and $Mg(NO_3)_2 \cdot 6H_2O$ (11.5 g, 44.9 mmol) were dissolved in 200 mL of 55:45 (v:v) methanol:*N,N*-dimethylformamide (DMF) using sonication. The solution was filtered through filter paper to remove any undissolved particulates and added to a 350-mL glass pressure vessel equipped with a stir bar. The reaction mixture was sparged with N_2 for 1 h. The reactor was sealed with a Teflon cap and heated in a silicone oil bath at 120 °C for 14 h with slow stirring (approximately 120 min^{-1} stirring rate). The non-homogenous mixture was filtered, and the solid was quickly transferred to a Pyrex jar containing 250 mL of *N,N*-dimethylformamide (DMF). The jar was placed in an oven that had been preheated to 60 °C and allowed to stand for at least 3 h. The jar was then allowed to cool to room temperature, and the solvent was decanted and replaced with 250 mL of fresh DMF. The jar was heated again to 60 °C, and this washing process was repeated two additional times. Next, the DMF was replaced with 250 mL of methanol, and the same washing process was repeated an additional three times using methanol to afford methanol-solvated Mg_2(dobpdc) as a white powder.

Synthesis of diamine-appended variants of Mg_2(dobpdc). Diamine-appended variants of Mg_2(dobpdc) were synthesized as described previously.[2] Although some previously published procedures[1] involved sonication of a slurry of Mg_2(dobpdc) in the diamine solution, no sonication was used during diamine grafting in this study so as to minimize possible variations in particle size among samples. Diamine loadings were determined by suspending ~5 mg of the diamine-appended metal–organic framework in 0.5 mL of DMSO-d_6, adding several drops of DCl (35 wt % in D_2O), heating until the mixture became homogeneous, and analyzing the resulting solution by ^{1}H NMR spectroscopy.

Thermogravimetric analysis. Thermogravimetric analysis (TGA) experiments were conducted using a TA Instruments TGA Q5000. Masses were uncorrected for buoyancy effects. Custom CO_2/N_2 blends (15% and 5% CO_2 in N_2) were purchased from Praxair. In all experiments, a flow rate of 25 mL/min was used, unless indicated otherwise. All experiments were performed at atmospheric pressure. Upon switching gas streams, a delay of 19 s was observed prior to the sample mass increasing, corresponding to the time required for CO_2 to reach the sample in the TGA furnace. Except where indicated otherwise, t = 0 corresponds to the time at which the gas stream within the TGA was switched (*i.e.*, 19 s before the new gas stream reached the sample). Adsorption isobars were performed as described previously,[3] with cooling ramp rates indicated in the figure legends. Adsorption kinetics experiments were performed by activating the sample under flowing N_2 for 15–30 min (at temperatures indicated in each figure legend) until the mass stabilized, followed by cooling to a temperature of interest, equilibration of the temperature, and switching the gas flow to a CO_2-containing stream. The sample was held isothermally until the mass stabilized, after which the sample was reactivated under N_2 (time and temperature specified in each figure legend), cooled to a new temperature of interest, then exposed again to the CO_2-containing stream. For plots in which kinetics experiments at multiple temperatures are presented for the same material, the exact same sample of the material was used for all temperatures (*i.e.*, the sample was re-activated and re-analyzed multiple times while keeping the same sample within the TGA furnace). Desorption kinetics of CO_2 under a dry N_2 stream were examined by activating adsorbents *in situ* in the TGA furnace as described above, exposing them to pure CO_2 at a sufficiently low temperature to reach full occupancy, heating the sample to the temperature of interest under pure CO_2, equilibrating the temperature, and finally switching the gas stream to dry N_2. The sample was held isothermally while the decrease in sample mass was monitored. The same sample was then exposed again to pure CO_2 to reach full occupancy, and a new desorption temperature was investigated using the same procedure.

Fitting TGA adsorption kinetics data to the Avrami or first-order model. In order to fit the data, the time points were normalized such that t = 0 corresponded to the data point immediately before mass increase was observed due to CO_2 adsorption (*i.e.*, t = 0 is after the 19-s delay prior to CO_2 reaching the sample in the furnace). Kinetic parameters (k_1 for pseudo-first order model; k_A and n_A for the Avrami model) were fit by minimizing the sum of the squared

residuals between the experimental (Q_{expt}) and predicted (Q_{pred}) occupancies, or $\sum(Q_{expt} - Q_{pred})^2$, using eqns (1) and (2). All adsorption experiments for which fits were prepared were run for a sufficiently long time to allow the amount of CO_2 adsorbed to stabilize, and the equilibrium occupancy Q_e was determined for each measurement by averaging the last 5 data points of the experiment.

Fitting Arrhenius plots with logarithmic functions. Logarithmic fits to the data in the Arrhenius plots of $\ln(k_A)$ *vs*. $1/T$ were functions of the form: $y = a + b \cdot \ln(x + c)$; see Figures S13–18. The parameters a, b, and c were fit by minimizing the sum of the squared residuals between the experimental and predicted $\ln(k_A)$ values.

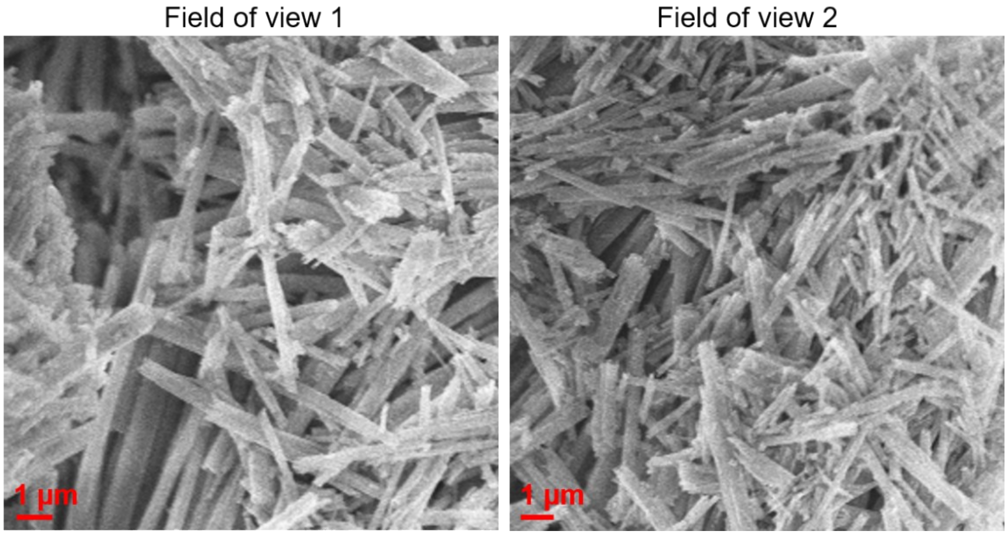

Fig. S1. Scanning electron microscopy images of Mg_2(dobpdc) used in this study. Two representative fields of view are shown. In general, the rod-shaped crystallites have an aspect ratio of approximately 10. The crystallites are heterogeneous in size, exhibiting widths ranging from ~60 nm to >1 μm. Samples were prepared for SEM and micrographs were recorded as described previously.[3]

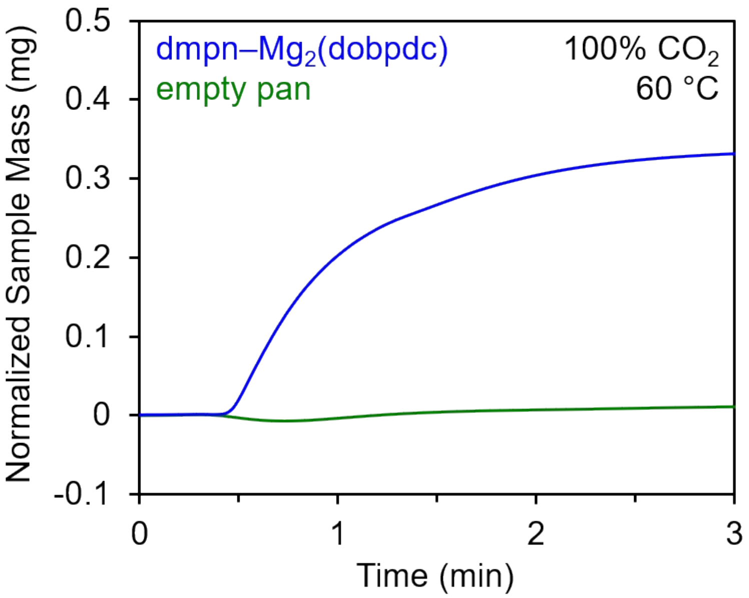

Fig. S2. Effect of buoyancy on apparent sample mass is negligible compared to the mass of CO_2 adsorbed by diamine–Mg_2(dobpdc). A flow rate of 25 mL/min was used in this experiment. An empty pan (green) or activated dmpn–Mg_2(dobpdc) (blue) was equilibrated at 60 °C under flowing N_2, and the gas flow was switched to CO_2. The mass of the activated dmpn–Mg_2(dobpdc) sample was 2.48 mg, and the amount of CO_2 adsorbed at t = 3 min was 0.33 mg, which corresponds to 13.4 g/100 g.

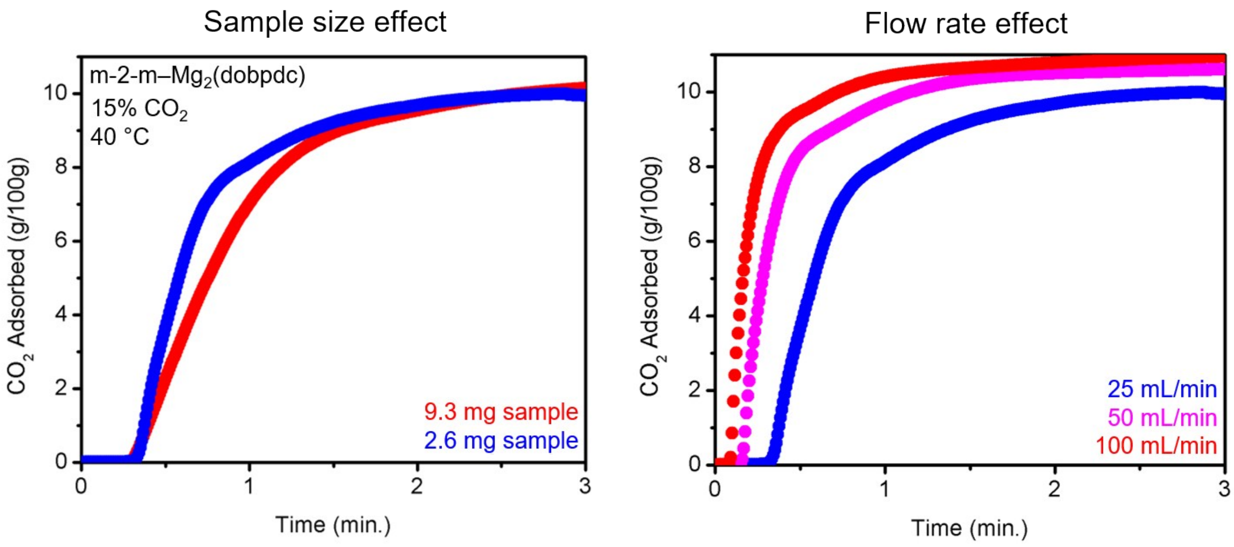

Fig. S3. Effect of sample size (left) and flow rate (right) on apparent adsorption kinetics in a thermogravimetric analysis (TGA) assay. Smaller sample masses and faster flow rates lead to faster apparent adsorption kinetics. These data were collected for m-2-m–Mg_2(dobpdc) at 40 °C from a 15% CO_2 stream. Note that faster flow rates lead to a shortening of the time required for CO_2 to reach the sample in the furnace, as expected.

Table S1: Delay Prior to Maximum Rate for CO_2 Adsorption by Bare Mg_2(dobpdc) from a 100% CO_2 Stream (Corresponding to Data in Fig. 3H in Main Text)

Temperature (°C)	Time of First Mass Increase (s)	Time at Which Maximum Rate Was Observed (s)	Delay Prior to Maximum Rate (s)
30	19	42	23
40	19	40	21
50	19	38	19
60	19	37	18
70	19	36	17
80	19	35	16
90	19	34	15
100	19	34	15
110	19	33	14
120	19	33	14

The data in this table correspond to Fig. 3H in the main text, which presents rate *vs.* time for CO_2 adsorption by bare Mg_2(dobpdc) from a 100% CO_2 stream at varying temperatures. The adsorbent was activated under N_2, cooled to the temperature of interest, and the gas stream was switched to 100% CO_2. As stated in the main text, CO_2 adsorption kinetics in this material are expected to be diffusion-limited. Therefore, any delay prior to reaching the maximum rate of CO_2 adsorption can be treated as a rough approximation of the time required for complete exchange of CO_2 for N_2 in the furnace atmosphere. The time at which the maximum rate was observed varied from 33–42 s, depending on the temperature. The sample mass did not begin increasing until 19 s, reflecting the time required for CO_2 to reach the sample within the furnace due to the design of the instrumentation. Therefore, the delay between CO_2 first reaching the sample and the time of maximum rate was 14–23 s, depending on the temperature. These delay times represent approximate lower bounds on the time required for the furnace atmosphere to change from 100% N_2 to 100% CO_2.

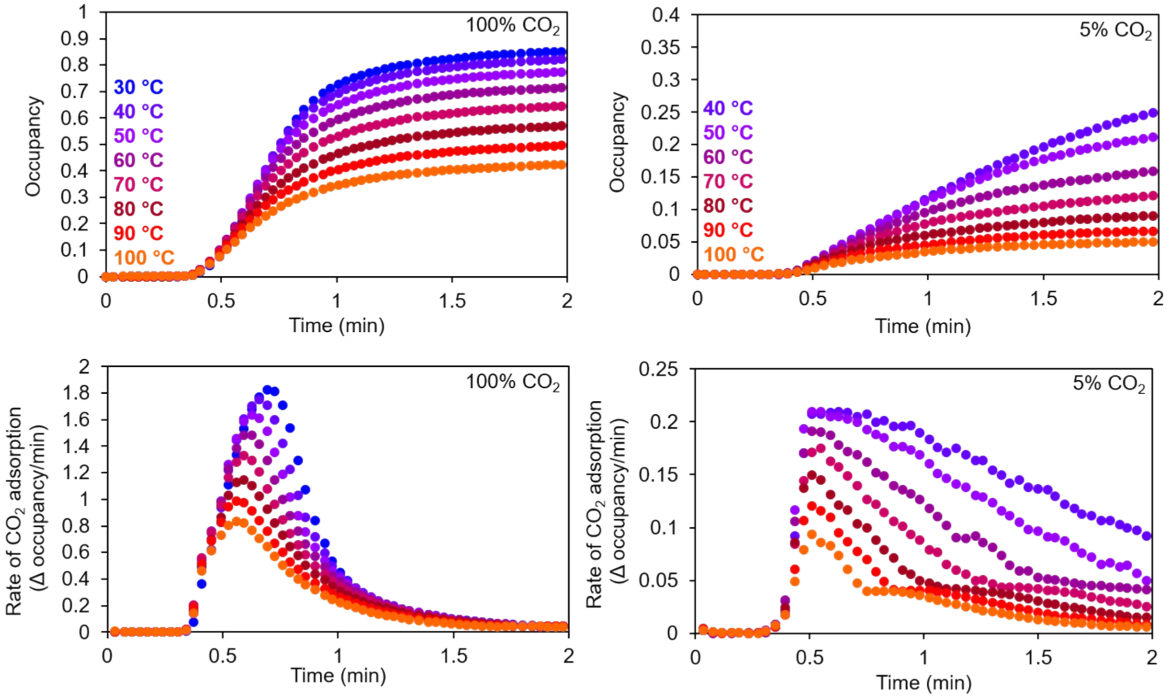

Fig. S4. Rate of CO_2 adsorption in bare Mg_2(dobpdc) from a 100% CO_2 stream (left column) or a 5% CO_2 stream (right column) at the temperatures indicated. Plots of occupancy *vs.* time are shown in the top row, and plots of adsorption rate *vs.* time are shown in the bottom row. Occupancy of 1.0 = 27.6 g/100 g.

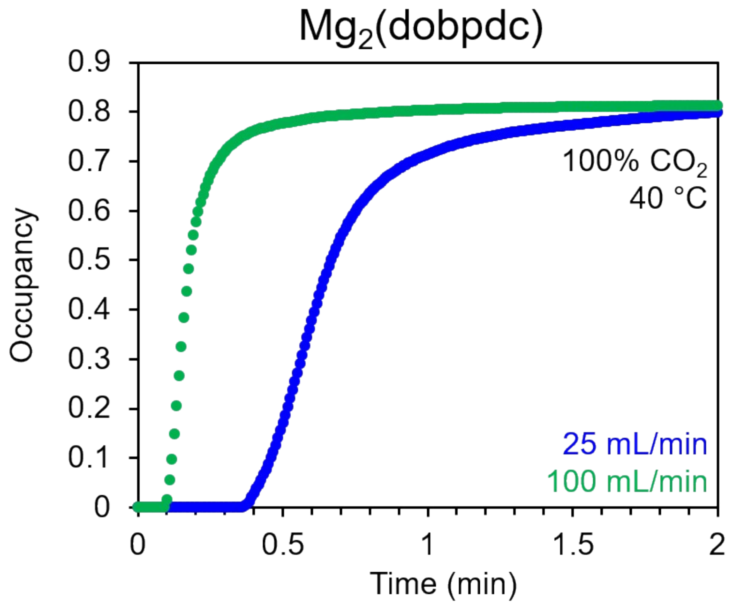

Fig. S5. Effect of TGA flow rate on CO_2 adsorption kinetics in bare Mg_2(dobpdc). Occupancy of 1.0 = 27.6 g/100 g. Note that faster flow rates lead to a shortening of the time required for CO_2 to reach the sample in the furnace, as expected.

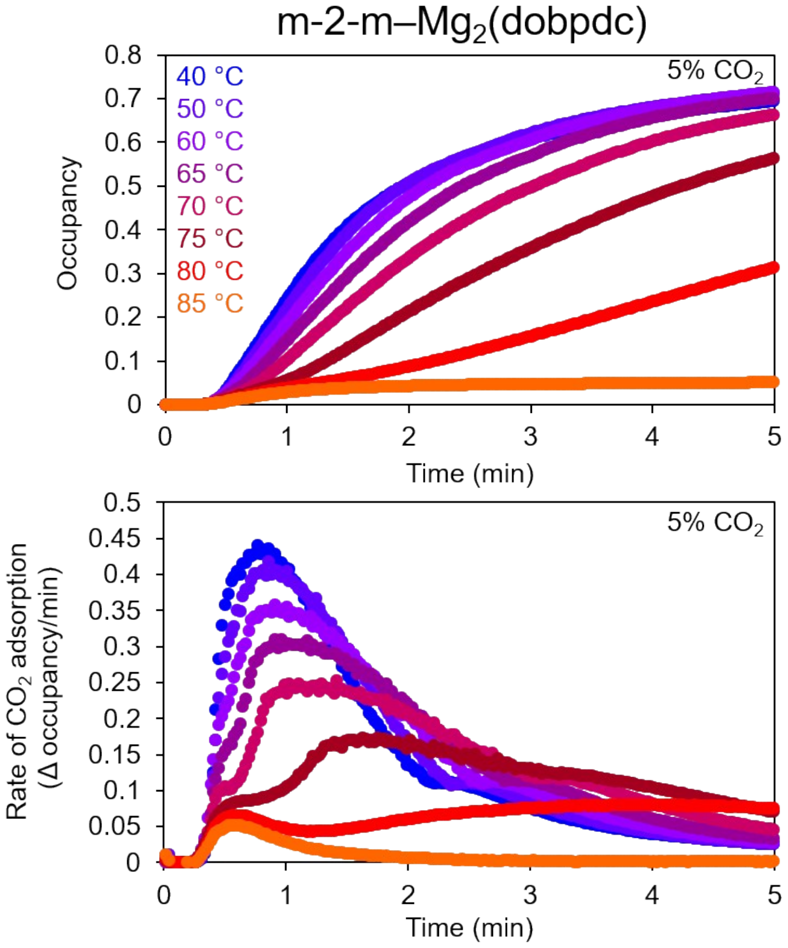

Fig. S6. Adsorption kinetics from a 5% CO_2 stream by m-2-m–Mg_2(dobpdc) at the temperatures indicated. The upper plot presents the fraction of CO_2 sites occupied *vs.* time, and the lower plot presents rate of CO_2 adsorption *vs.* time. Occupancy of 1.0 = 17.8 g/100 g.

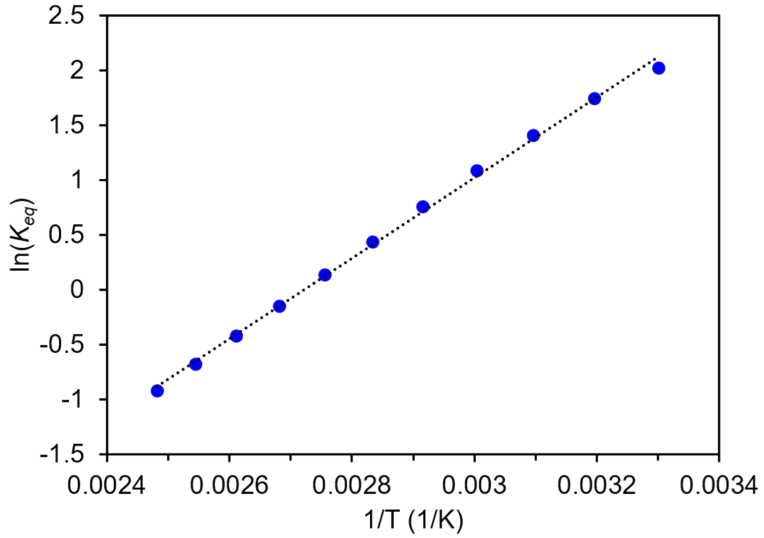

Fig. S7. Van't Hoff plot depicting the equilibrium CO_2 adsorption capacity with varying temperature for Mg_2(dobpdc) from a 100% CO_2 stream. These are the same data depicted in Fig. 4 in the main text, corresponding to temperatures from 30 to 130 °C. A linear fit is included (R^2 = 0.998). An estimation of Δh of CO_2 adsorption in bare Mg_2(dobpdc) can be made based on the assumption that the slope of the linear fit equals $-\Delta h/R$. When the data points corresponding to the temperatures 30–130 °C are included, this analysis yields $\Delta h = -30.5$ kJ/mol. These values are slightly different from the Δh values previously reported for this material based on CO_2 adsorption isotherms: approximately −44 kJ/mol at low loadings and approximately −40 kJ/mol at a loading of 3.5 mmol/g.[4] This discrepancy may be due to N_2 being bound to a small fraction of the metal sites prior to CO_2 exposure in the TGA experiment.[5] In contrast, in the previously reported gas adsorption isotherms, CO_2 was dosed into a sample that had been activated under vacuum (*i.e.*, no N_2 was present).[4]

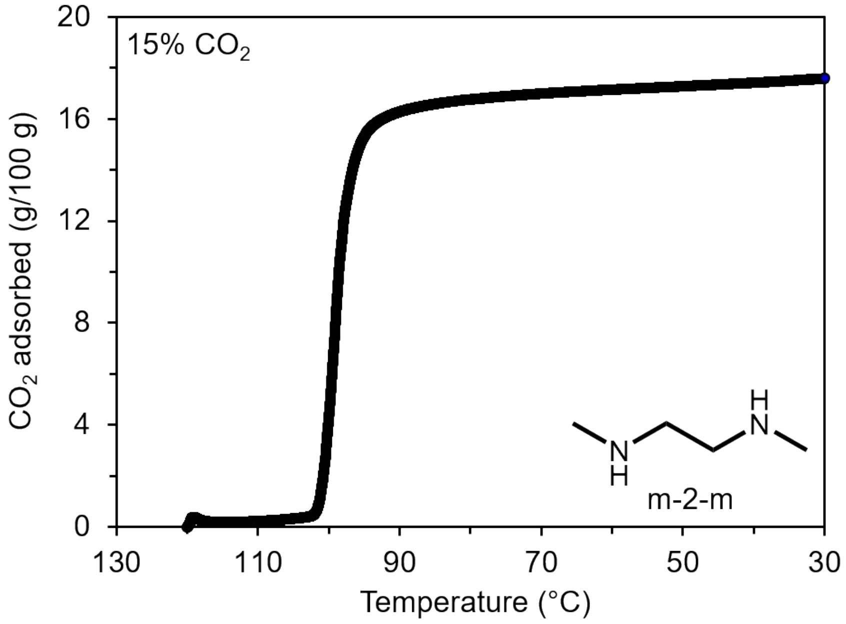

Fig. S8. 15% CO_2 adsorption isobar for m-2-m–Mg_2(dobpdc). A ramp rate of 0.5 °C/min was used. The onset of the CO_2 adsorption step is at 102 °C.

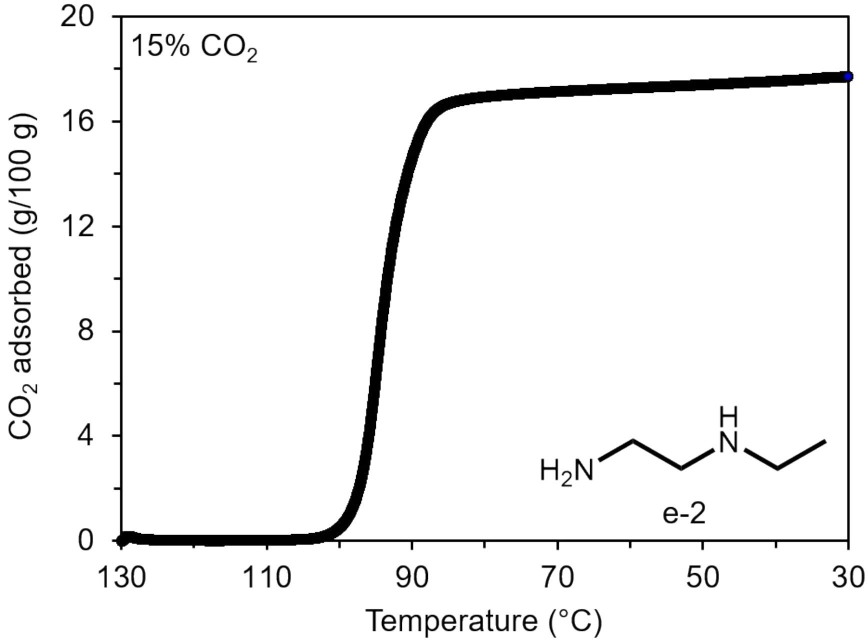

Fig. S9. 15% CO_2 adsorption isobar for e-2–Mg_2(dobpdc). A ramp rate of 0.5 °C/min was used. The onset of the CO_2 adsorption step is at 98 °C.

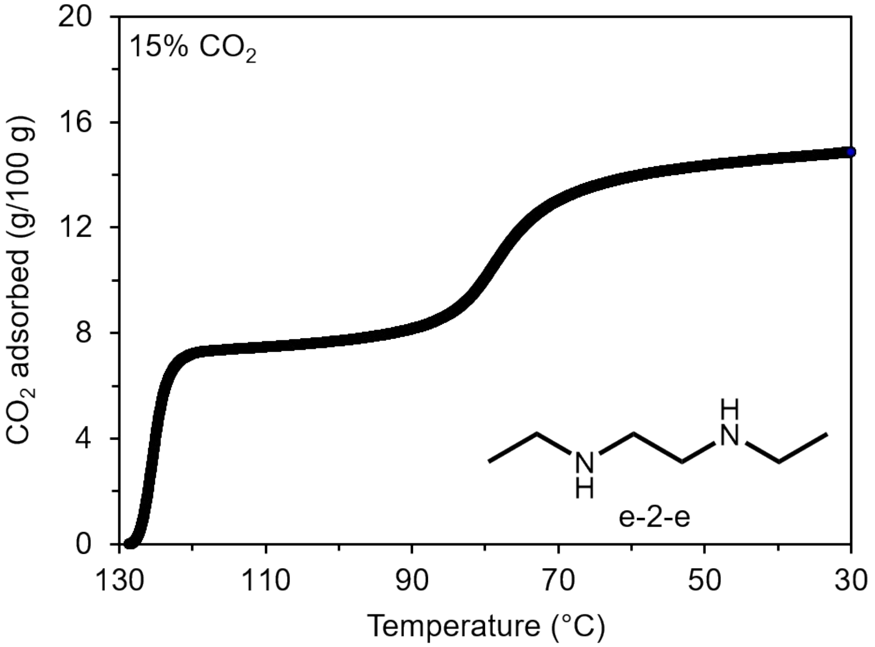

Fig. S10. 15% CO_2 adsorption isobar for e-2-e–Mg_2(dobpdc). A ramp rate of 0.5 °C/min was used. The onset of the CO_2 adsorption step is at 128 °C. As noted in the main text, this material exhibits a two-step adsorption profile, and Fig. 9 only depicts temperatures under which the low-temperature step was not operative for this material.

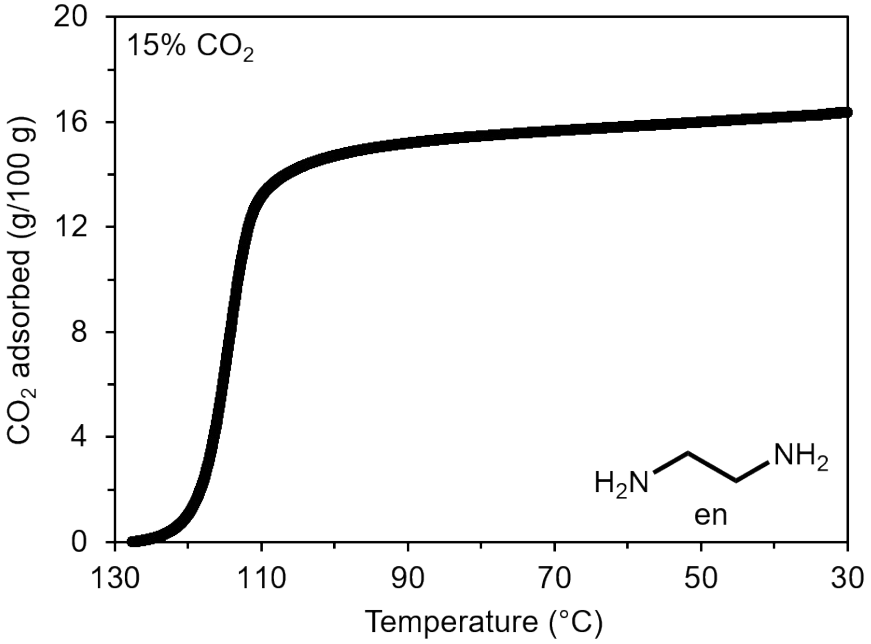

Fig. S11. 15% CO_2 adsorption isobar for en–Mg_2(dobpdc). A ramp rate of 0.5 °C/min was used. The onset of the CO_2 adsorption step is at 120 °C.

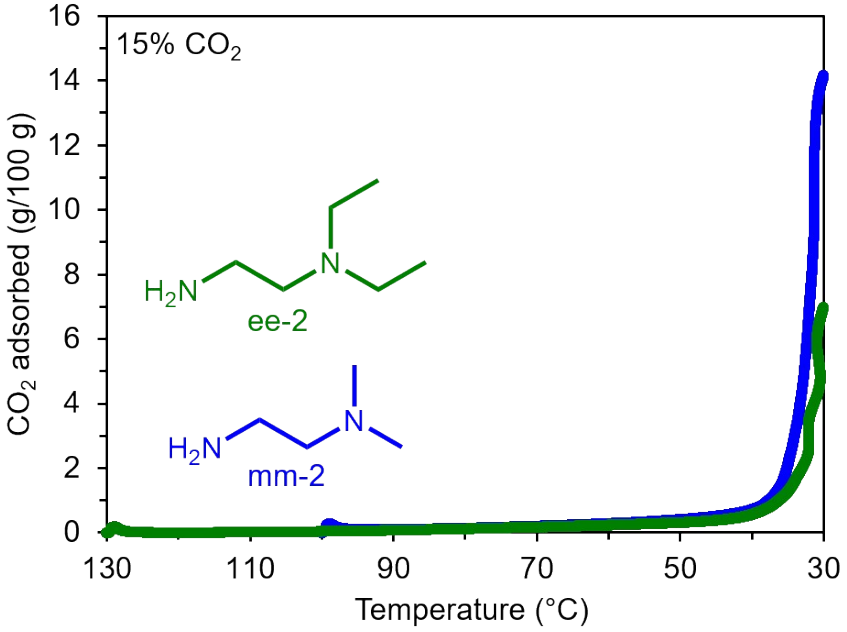

Fig. S12. 15% CO_2 adsorption isobars for ee-2–Mg_2(dobpdc) and mm-2–Mg_2(dobpdc). A ramp rate of 0.5 °C/min was used in both experiments. Minimal uptake of CO_2 was observed in both materials at temperatures above 40 °C.

Table S2: Comparison of 15% CO_2 Adsorption Step Positions Determined from Isobars or Linear Fits of k_A *vs.* T

Diamine-Appended Mg_2(dobpdc) Variant*	T_{step} (°C) Determined from Isobars	T_{step} (°C) Determined from *x*-Intercepts of Linear k_A *vs.* T Plots
m-2-m	102	100
e-2-e	128	127
m-2	N.D.	102
e-2	98	96
i-2	N.D.	100
en	120	117

*Note: Data are not included for ±-*trans*-dach–Mg_2(dobpdc) because T_{step} could not be determined from the k_A *vs.* T plot; see Fig. S20.

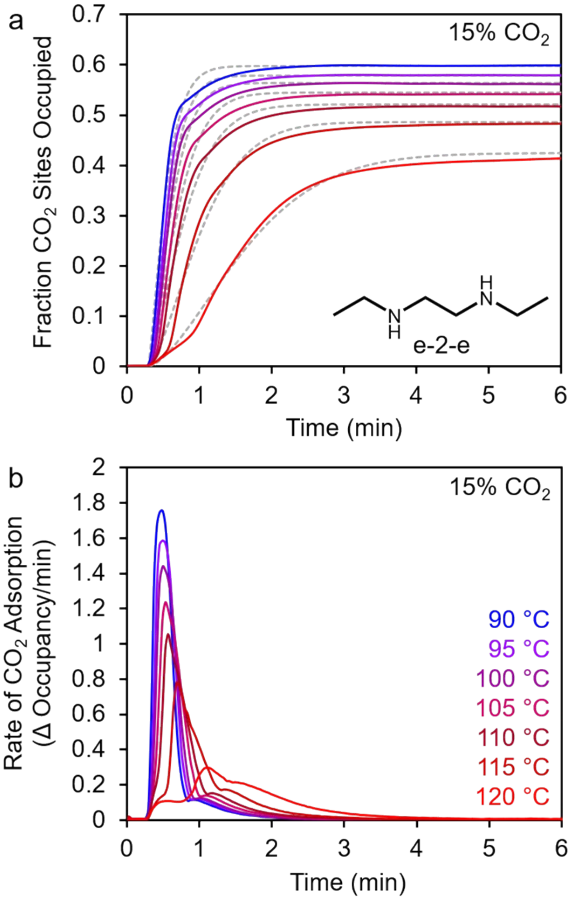

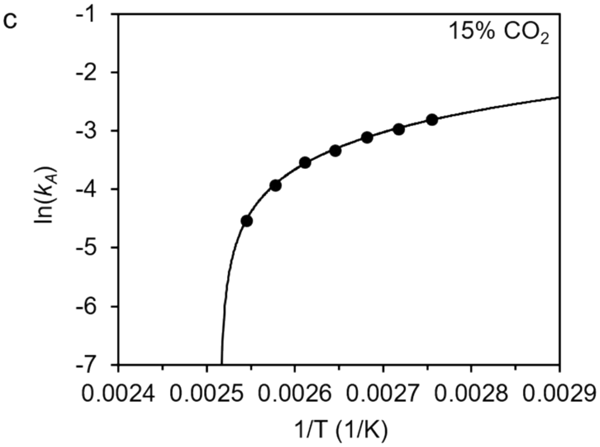

d

T (°C)	k_A (s^{-1})	n_A	R^2 value (Avrami fit)	Occupancy at equilibrium	CO_2 Capacity at equilibrium (g/100 g)
90	0.0609	1.26	0.989	0.597	9.56
95	0.0511	1.39	0.988	0.579	9.27
100	0.0449	1.40	0.988	0.563	9.01
105	0.0359	1.38	0.988	0.544	8.71
110	0.0291	1.35	0.988	0.522	8.35
115	0.0198	1.48	0.991	0.486	7.78
120	0.0108	1.56	0.993	0.425	6.79

Fig. S13. Adsorption of CO_2 from a 15% stream by e-2-e–Mg_2(dobpdc). (a) Adsorption *vs.* time. The solid lines represent experimental data at the temperatures indicated by color-coding, and the gray dashed lines represent fits to the data using the Avrami equation. Sample activation: 130 °C for 5 min under N_2. The maximum adsorption capacity assuming one CO_2 per diamine is 16.0 g/100 g. (b) Rate of CO_2 adsorption *vs.* time corresponding to the data shown in (a). (c) Arrhenius plot for the Avrami rate constants (k_A) corresponding to the fits shown in (a). The curved line represents a logarithmic fit to the data: $y = 3.95 + 0.812*\ln(x - 0.00252)$. Root mean square error (RMSE): 0.016. Range of the dependent variable: 1.72. RMSE / range = 0.0091. (d) Adsorption capacities and kinetic parameters corresponding to the data and fits shown in (a).

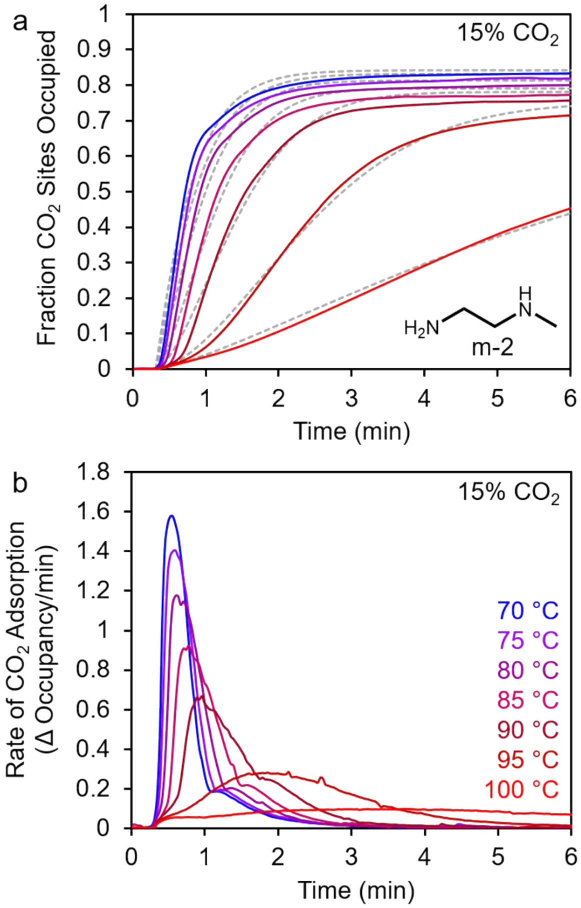

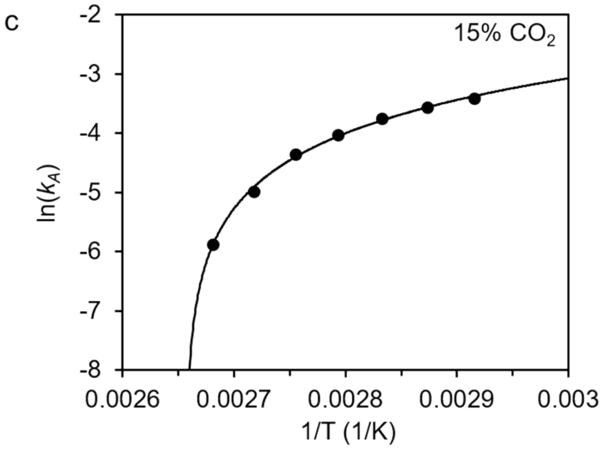

d

T (°C)	k_A (s^{-1})	n_A	R^2 value (Avrami fit)	Occupancy at equilibrium	CO_2 Capacity at equilibrium (g/100 g)
70	0.0328	1.07	0.981	0.840	16.0
75	0.0283	1.14	0.979	0.831	15.9
80	0.0234	1.32	0.983	0.814	15.6
85	0.0178	1.49	0.985	0.789	15.1
90	0.0128	1.59	0.984	0.782	14.9
95	0.0068	1.69	0.995	0.752	14.4
100	0.0028	1.32	0.998	0.715	13.7

Fig. S14. Adsorption of CO_2 from a 15% stream by m-2–Mg_2(dobpdc). (a) Adsorption *vs.* time. The solid lines represent experimental data at the temperatures indicated by color-coding, and the gray dashed lines represent fits to the data using the Avrami equation. Sample activation: 130 °C for 5 min under N_2. The maximum adsorption capacity assuming one CO_2 per diamine is 18.8 g/100 g. (b) Rate of CO_2 adsorption *vs.* time corresponding to the data shown in (a). (c) Arrhenius plot for the Avrami rate constants (k_A) corresponding to the fits shown in (a). The curved line represents a logarithmic fit to the data: $y = 5.39 + 1.06*\ln(x - 0.00266)$. Root mean square error (RMSE): 0.039. Range of the dependent variable: 2.51. RMSE / range = 0.016. (d) Adsorption capacities and kinetic parameters corresponding to the data and fits shown in (a).

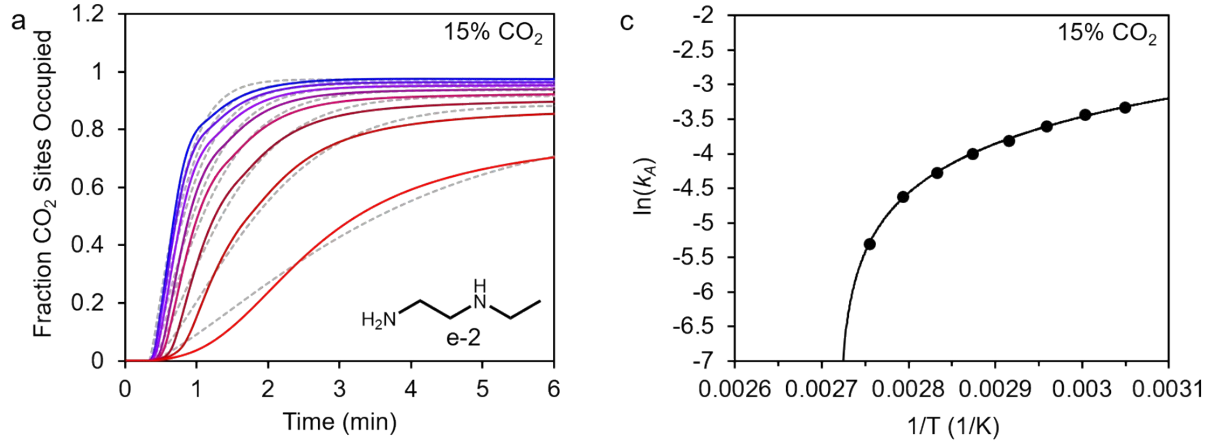

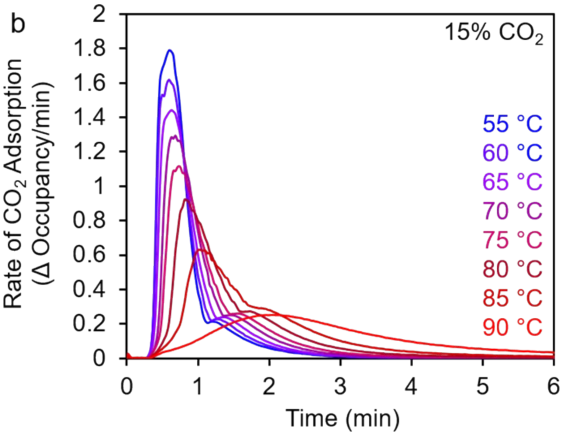

d

T (°C)	k_A (s^{-1})	n_A	R^2 value (Avrami fit)	Occupancy at equilibrium	CO_2 Capacity at equilibrium (g/100 g)
55	0.0361	1.24	0.993	0.974	17.3
60	0.0323	1.19	0.994	0.966	17.2
65	0.0272	1.21	0.993	0.959	17.1
70	0.0221	1.28	0.991	0.949	16.9
75	0.0183	1.27	0.990	0.936	16.7
80	0.0139	1.36	0.989	0.916	16.3
85	0.0099	1.42	0.990	0.886	15.8
90	0.0050	1.31	0.986	0.818	14.6

Fig. S15. Adsorption of CO_2 from a 15% stream by e-2–Mg_2(dobpdc). (a) Adsorption *vs.* time. The solid lines represent experimental data at the temperatures indicated by color-coding, and the gray dashed lines represent fits to the data using the Avrami equation. Sample activation: 130 °C for 5 min under N_2. The maximum adsorption capacity assuming one CO_2 per diamine is 17.8 g/100 g. (b) Rate of CO_2 adsorption *vs.* time corresponding to the data shown in (a). (c) Arrhenius plot for the Avrami rate constants (k_A) corresponding to the fits shown in (a). The curved line represents a logarithmic fit to the data: $y = 3.76 + 0.883*\ln(x - 0.00272)$. Root mean square error (RMSE): 0.018. Range of the dependent variable: 1.97. RMSE / range = 0.0089. (d) Adsorption capacities and kinetic parameters corresponding to the data and fits shown in (a).

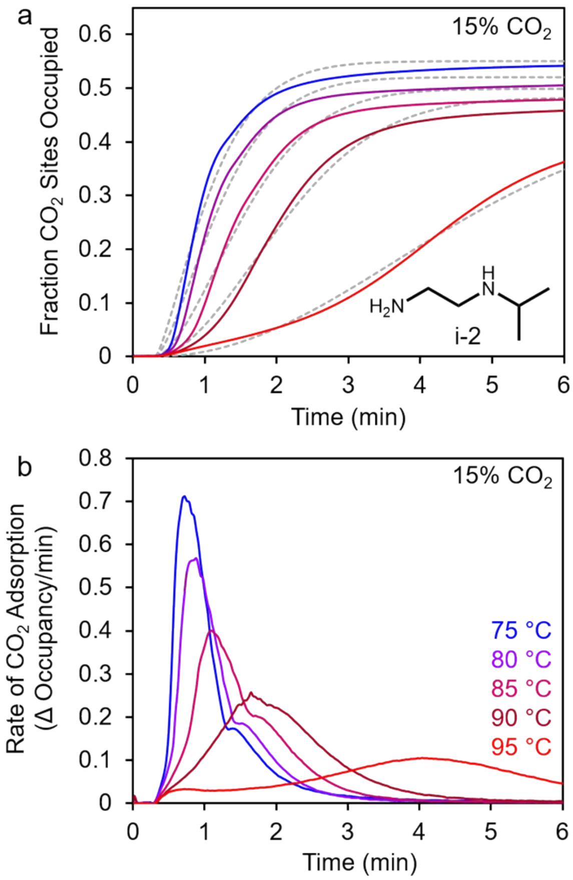

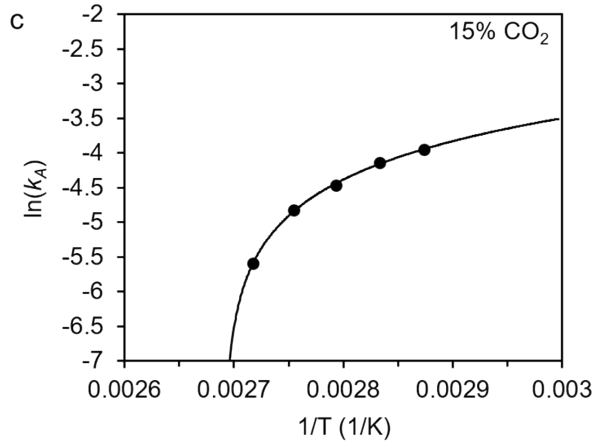

d

T (°C)	k_A (s^{-1})	n_A	R^2 value (Avrami fit)	Occupancy at equilibrium	CO_2 Capacity at equilibrium (g/100 g)
75	0.0193	1.34	0.984	0.551	9.3
80	0.0159	1.47	0.982	0.520	8.7
85	0.0115	1.65	0.982	0.499	8.4
90	0.0080	1.83	0.985	0.483	8.1
95	0.0037	2.04	0.994	0.449	7.5

Fig. S16. Adsorption of CO_2 from a 15% stream by i-2–Mg_2(dobpdc). (a) Adsorption *vs.* time. The solid lines represent experimental data at the temperatures indicated by color-coding, and the gray dashed lines represent fits to the data using the Avrami equation. Sample activation: 130 °C for 5 min under N_2. The maximum adsorption capacity assuming one CO_2 per diamine is 16.8 g/100 g. (b) Rate of CO_2 adsorption *vs.* time corresponding to the data shown in (a). (c) Arrhenius plot for the Avrami rate constants (k_A) corresponding to the fits shown in (a). The curved line represents a logarithmic fit to the data: $y = 3.35 + 0.848*\ln(x - 0.00269)$. Root mean square error (RMSE): 0.016. Range of the dependent variable: 1.64. RMSE / range = 0.0098. (d) Adsorption capacities and kinetic parameters corresponding to the data and fits shown in (a).

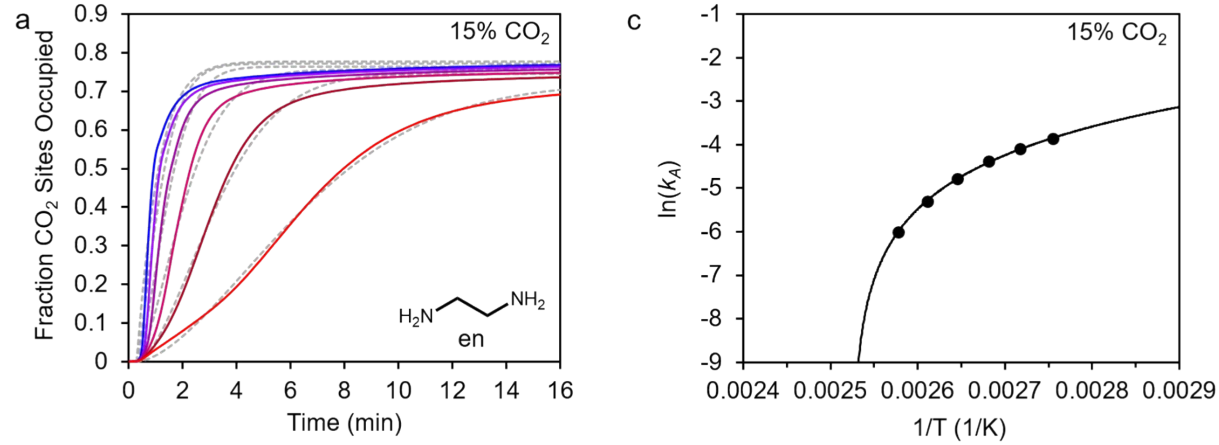

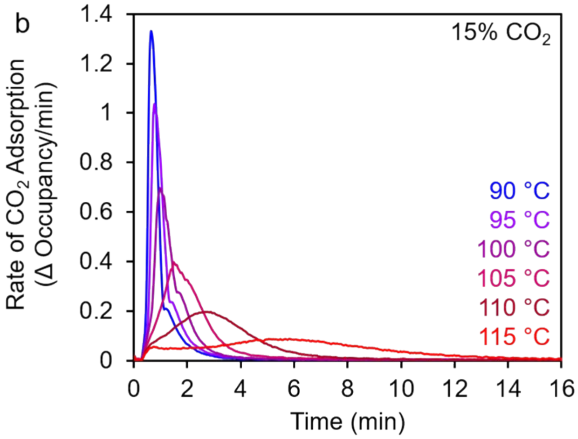

d

T (°C)	k_A (s^{-1})	n_A	R^2 value (Avrami fit)	Occupancy at equilibrium	CO_2 Capacity at equilibrium (g/100 g)
90	0.0211	1.07	0.946	0.777	15.5
95	0.0166	1.34	0.962	0.771	15.4
100	0.0124	1.52	0.977	0.764	15.3
105	0.0080	1.52	0.985	0.756	15.1
110	0.0048	1.67	0.994	0.745	14.9
115	0.0023	1.64	0.998	0.722	14.4

Fig. S17. Adsorption of CO_2 from a 15% stream by en–Mg_2(dobpdc). (a) Adsorption *vs.* time. The solid lines represent experimental data at the temperatures indicated by color-coding, and the gray dashed lines represent fits to the data using the Avrami equation. Sample activation: 130 °C for 5 min under N_2. The maximum adsorption capacity assuming one CO_2 per diamine is 20.0 g/100 g. (b) Rate of CO_2 adsorption *vs.* time corresponding to the data shown in (a). (c) Arrhenius plot for the Avrami rate constants (k_A) corresponding to the fits shown in (a). The curved line represents a logarithmic fit to the data: $y = 8.35 + 1.45*\ln(x - 0.00253)$. Root mean square error (RMSE): 0.014. Range of the dependent variable: 2.16. RMSE / range = 0.0063. (d) Adsorption capacities and kinetic parameters corresponding to the data and fits shown in (a).

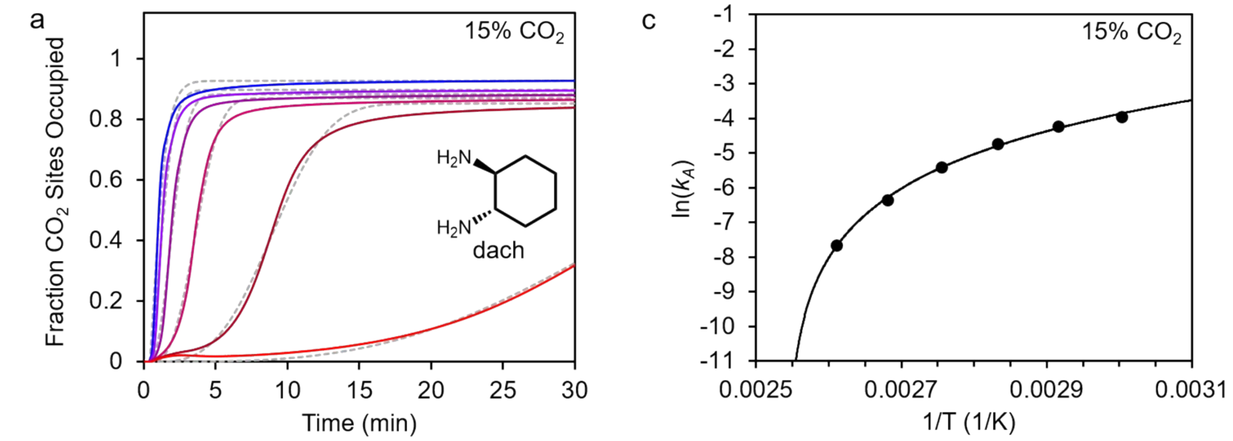

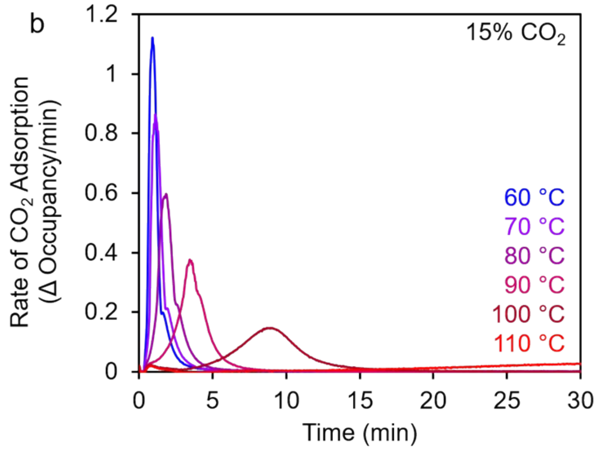

d

T (°C)	k_A (s^{-1})	n_A	R^2 value (Avrami fit)	Occupancy at equilibrium	CO_2 Capacity at equilibrium (g/100 g)
60	0.0194	1.27	0.977	0.927	14.9
70	0.0146	1.63	0.988	0.897	14.4
80	0.0089	2.18	0.991	0.882	14.2
90	0.0045	2.84	0.993	0.869	14.0
100	0.0017	3.21	0.994	0.852	13.7
110	0.0005	3.16	0.999	0.762	12.3

Fig. S18. Adsorption of CO_2 from a 15% stream by ±-*trans*-dach–Mg_2(dobpdc). (a) Adsorption *vs.* time. The solid lines represent experimental data at the temperatures indicated by color-coding, and the gray dashed lines represent fits to the data using the Avrami equation. Sample activation: 150 °C for 10 min under N_2. The maximum adsorption capacity assuming one CO_2 per diamine is 16.1 g/100 g. (b) Rate of CO_2 adsorption *vs.* time corresponding to the data shown in (a). (c) Arrhenius plot for the Avrami rate constants (k_A) corresponding to the fits shown in (a). (c) Arrhenius plot for the Avrami rate constants (k_A) corresponding to the fits shown in (a). The curved line represents a logarithmic fit to the data: $y = 11.7 + 2.03*\ln(x - 0.00254)$. Root mean square error (RMSE): 0.060. Range of the dependent variable: 3.83. RMSE / range = 0.016. (d) Adsorption capacities and kinetic parameters corresponding to the data and fits shown in (a).

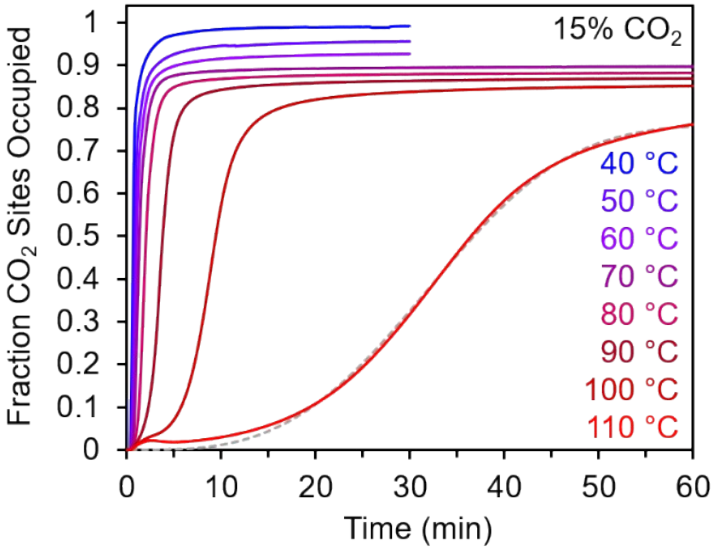

Fig. S19. Plot of CO_2 adsorption from a 15% CO_2 gas stream (balance N_2) *vs.* time by ±-*trans*-dach–Mg_2(dobpdc), showing a longer time-scale on the *x*-axis and more temperature conditions compared to Fig. S18. This diamine variant exhibited an unusually long induction period. The gray dotted line represents a fit to the 110 °C data using the Avrami equation (n_A = 3.16). Sample activation: 150 °C for 10 min under N_2. "Fraction CO_2 sites occupied" indicates the ratio of adsorbed CO_2 molecules to dach molecules in the material. The maximum adsorption capacity assuming one CO_2 per diamine is 16.1 g/100 g.

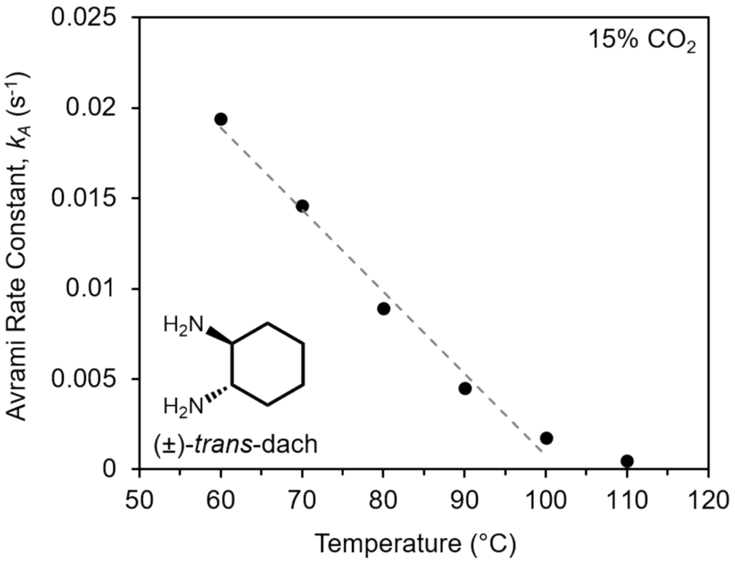

Fig. S20. Plot of Avrami rate constant (k_A) *vs.* temperature for CO_2 adsorption from a 15% stream in ±-*trans*-dach–Mg_2(dobpdc). The data points corresponding to 60–100 °C followed a linear trend, but the 110 °C data point—which was clearly below the step temperature based on the data shown in Figs. S18–19—deviated from this trend.

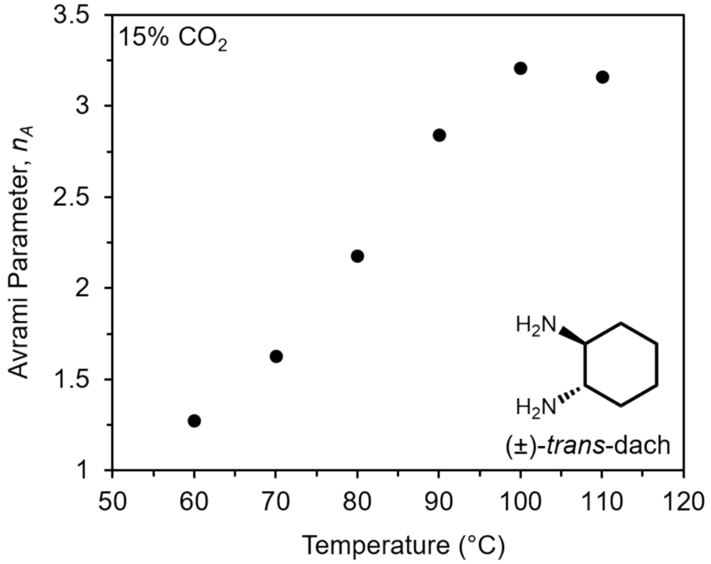

Fig. S21. Plot of Avrami rate parameter (n_A) *vs.* temperature for CO_2 adsorption from a 15% stream in ±-*trans*-dach–Mg_2(dobpdc).

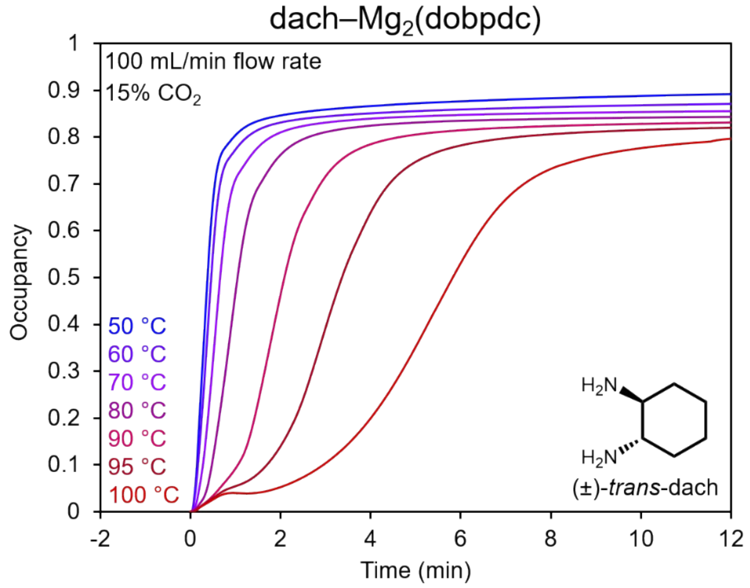

Fig. S22. The induction period for CO_2 adsorption from a 15% stream in dach–Mg_2(dobpdc) can be reduced by using faster flower rates. However, sigmoidal kinetics are observed even when a flow rate of 100 mL/min is used.

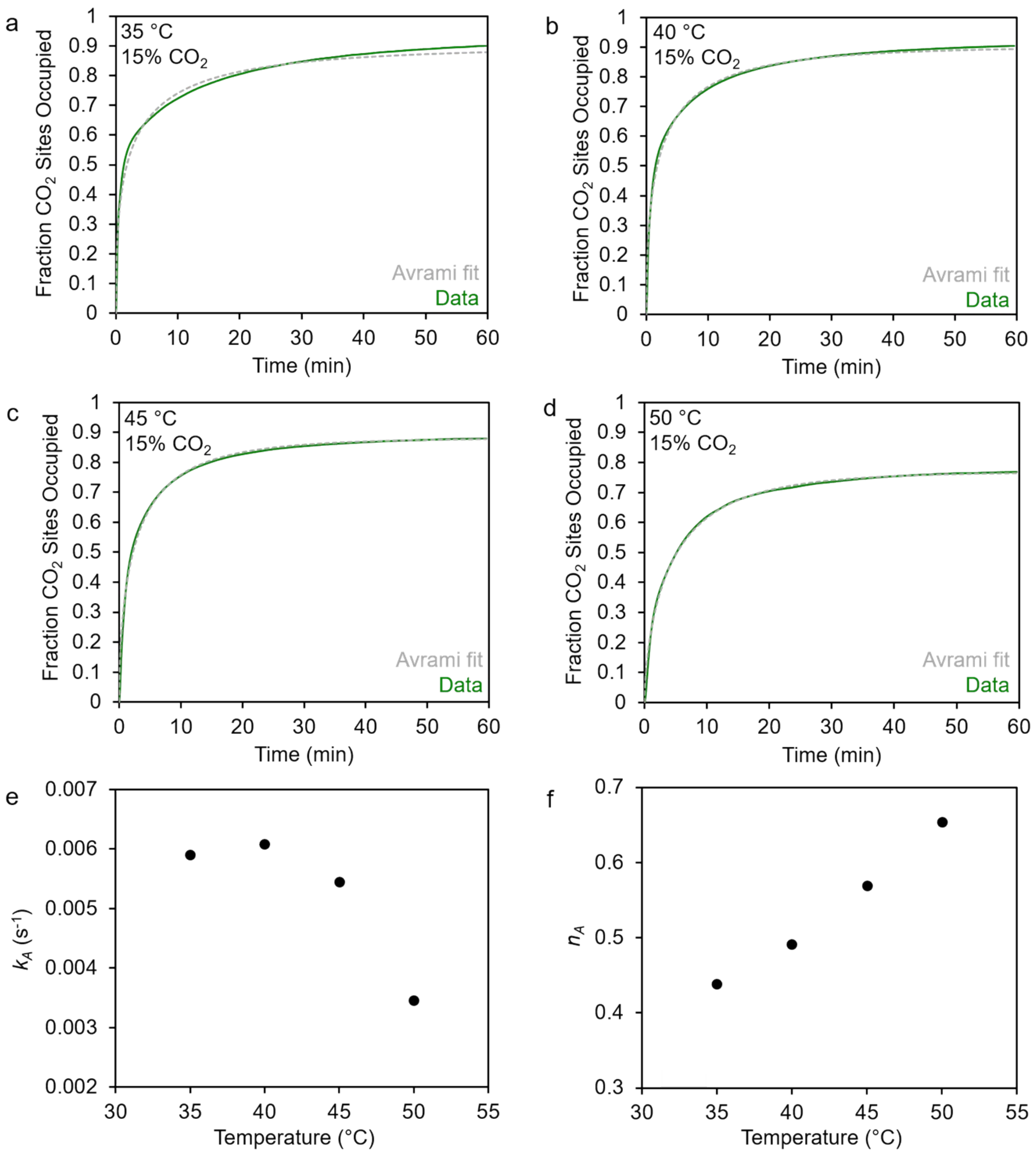

Fig. S23. Avrami model fits for CO_2 adsorption from a 15% stream by dmpn–Mg_2(dobpdc) from a 15% CO_2 stream; these are the same data as in Fig. 10 in the main text. Panels a, b, c, and d present Avrami fits overlaid with experimental data for adsorption at 35, 40, 45, and 50 °C, respectively. Panels e and f present the Avrami rate constant (k_A) and Avrami parameter (n_A) *vs.* temperature, respectively, derived from the data presented in panels a–d. For the 55 °C adsorption experiment, the mass had not equilibrated after 60 minutes, so the Avrami model was not applied.

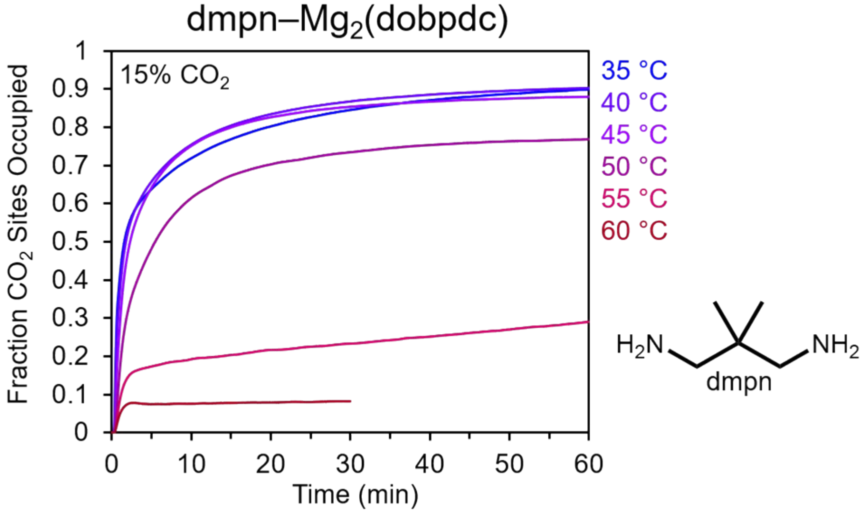

Fig. S24. Adsorption kinetics for dmpn–Mg_2(dobpdc) from a 15% CO_2 stream; these are the same data as in Fig. 10 in the main text, except a longer time scale is shown in the *x*-axis. For the 55 °C adsorption experiment, the mass had not equilibrated after 60 minutes.

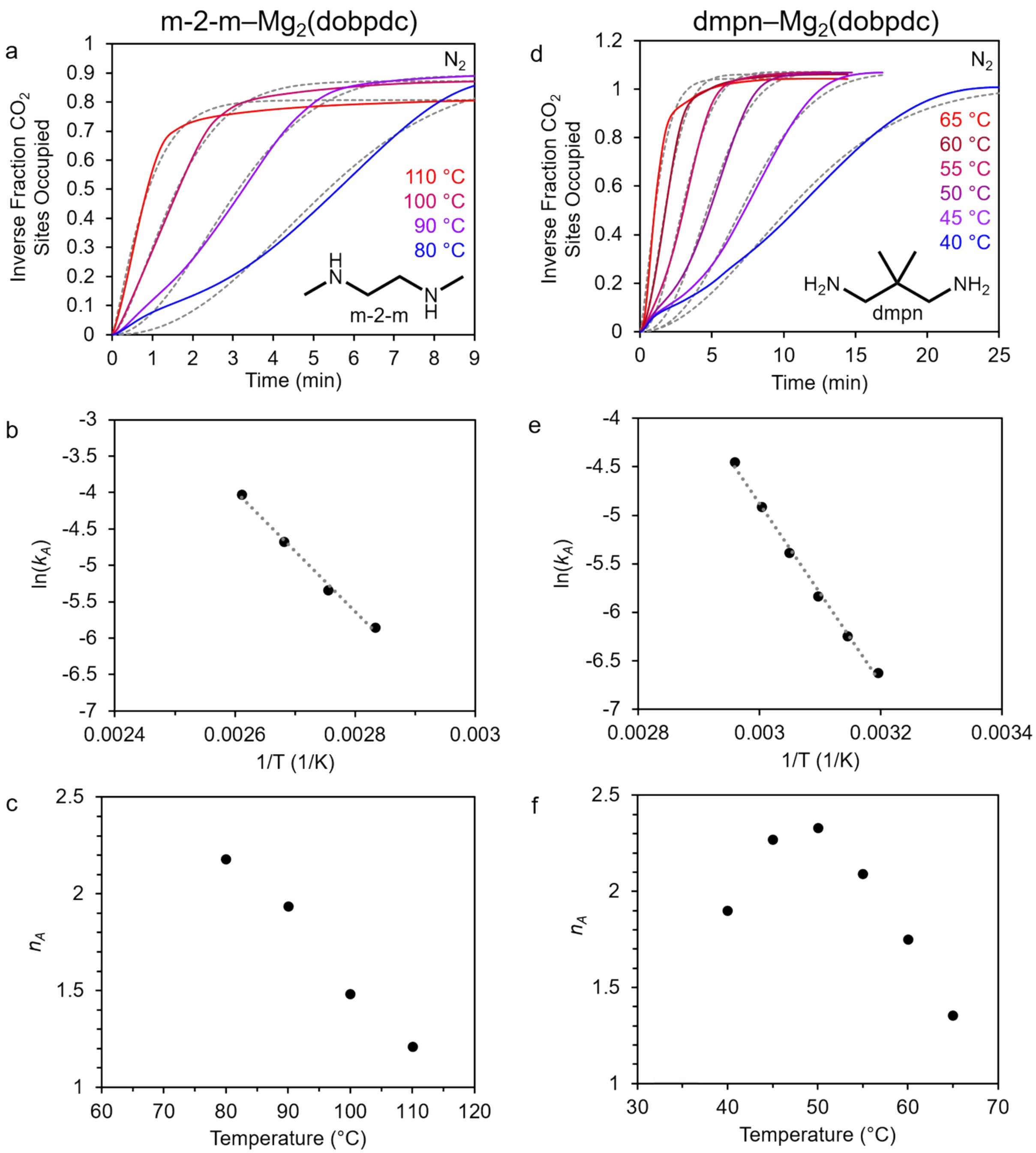

Fig. S25. Avrami fits to dry N_2 desorption kinetic data for m-2-m–Mg_2(dobpdc) and dmpn–Mg_2(dobpdc), corresponding to the data shown in Fig. 11 in the main text. Data are presented as "inverse occupancy" to facilitate visualization of the induction period characteristic of the Avrami model. In these experiments, equilibrium adsorption was reached under a 100% CO_2 stream at each indicated temperature, and the gas stream was switched to dry N_2 to initiate desorption. (a) Kinetics of CO_2 desorption from m-2-m–Mg_2(dobpdc) under a dry N_2 stream. The *y*-axis, "Inverse Fraction CO_2 Sites Occupied," is defined as $Q_i - Q_t$, where Q_i is the initial CO_2 capacity before the introduction of N_2. The solid lines represent experimental data at the temperatures indicated, and

the gray dashed lines represent fits to the data using the Avrami equation. (b) Arrhenius plot for the Avrami rate constants (k_A) corresponding to the fits shown in (a). The data exhibit normal Arrhenius behavior. (c) Plot of the Avrami parameter, n_A, *vs.* temperature, corresponding to the fits shown in (a). (d) Kinetics of CO_2 desorption from dmpn–Mg_2(dobpdc) under a dry N_2 stream. The *y*-axis is as defined in (a). The solid lines represent experimental data at the temperatures indicated by color-coding, and the gray dashed lines represent fits to the data using the Avrami equation. (e) Arrhenius plot for the Avrami rate constants (k_A) corresponding to the fits shown in (d). The data follow Arrhenius behavior. (f) Plot of the Avrami parameter, n_A, *vs.* temperature, corresponding to the fits shown in (d).

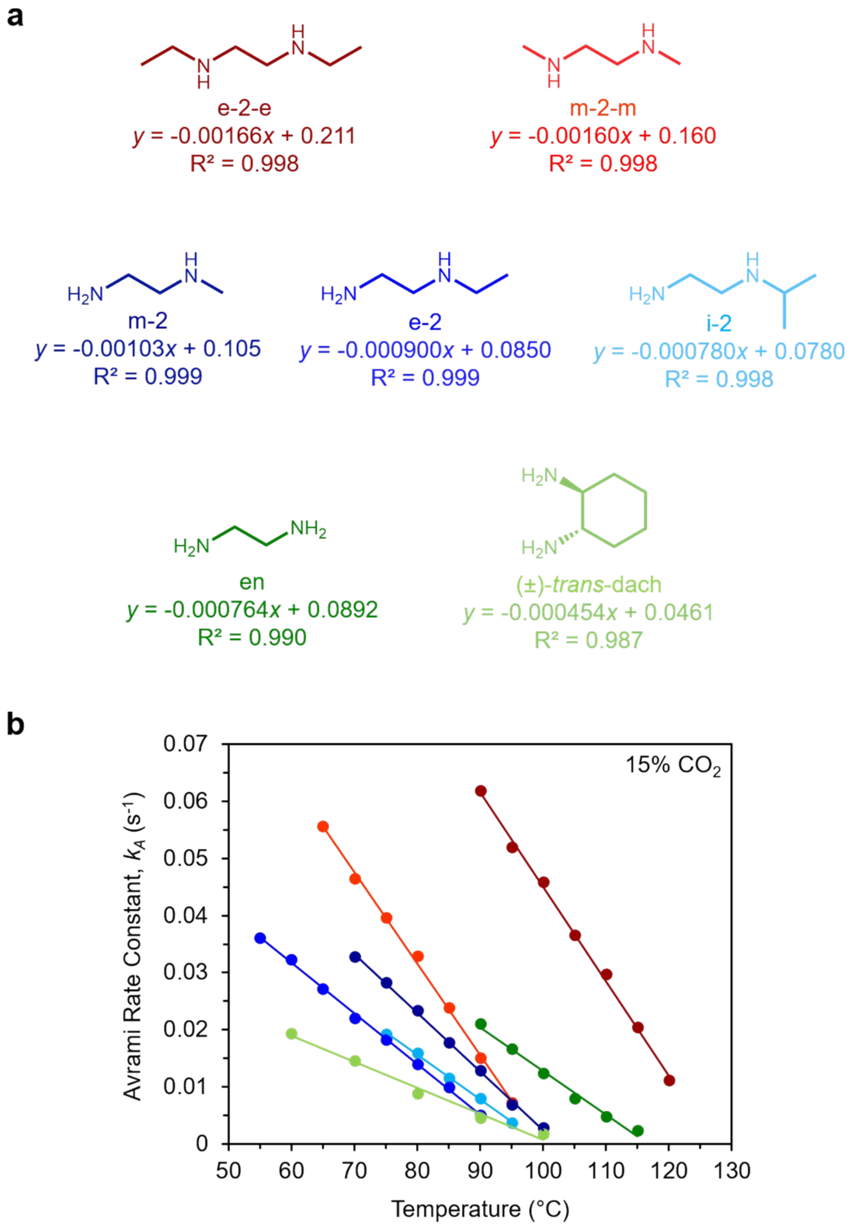

Fig. S26. Linear regressions for rate constants and Avrami parameters for 15% CO_2 adsorption by diamine-appended Mg_2(dobpdc) variants. Panels a and b here are the same as Fig. 9a and b, respectively, in the main text, except linear regression data are included here in panel a.

References

(1) Siegelman, R. L.; McDonald, T. M.; Gonzalez, M. I.; Martell, J. D.; Milner, P. J.; Mason, J. A.; Berger, A. H.; Bhown, A. S.; Long, J. R. Controlling Cooperative CO_2 Adsorption in Diamine-Appended Mg_2(dobpdc) Metal–Organic Frameworks. *J. Am. Chem. Soc.* **2017**, *139* (30), 10526–10538.

(2) Milner, P. J.; Siegelman, R. L.; Forse, A. C.; Gonzalez, M. I.; Runčevski, T.; Martell, J. D.; Reimer, J. A.; Long, J. R. A Diaminopropane-Appended Metal–Organic Framework Enabling Efficient CO_2 Capture from Coal Flue Gas via a Mixed Adsorption Mechanism. *J. Am. Chem. Soc.* **2017**, *139* (38), 13541–13553.

(3) Martell, J. D.; Porter-Zasada, L. B.; Forse, A. C.; Siegelman, R. L.; Gonzalez, M. I.; Oktawiec, J.; Runčevski, T.; Xu, J.; Srebro-Hooper, M.; Milner, P. J.; et al. Enantioselective Recognition of Ammonium Carbamates in a Chiral Metal–Organic Framework. *J. Am. Chem. Soc.* **2017**, *139* (44), 16000–16012.

(4) McDonald, T. M.; Lee, W. R.; Mason, J. A.; Wiers, B. M.; Hong, C. S.; Long, J. R. Capture of Carbon Dioxide from Air and Flue Gas in the Alkylamine-Appended Metal–Organic Framework Mmen-Mg_2(dobpdc). *J. Am. Chem. Soc.* **2012**, *134* (16), 7056–7065.

(5) Mason, J. A.; Sumida, K.; Herm, Z. R.; Krishna, R.; Long, J. R. Evaluating Metal–Organic Frameworks for Post-Combustion Carbon Dioxide Capture via Temperature Swing Adsorption. *Energy Environ. Sci.* **2011**, *4* (8), 3030–3040.
